# Varietal Differences in Kidney Beans Modulate Gut Microbiota and Inflammation During High-Fat Diet-Induced Obesity in Male Mice

**DOI:** 10.3390/nu18030461

**Published:** 2026-01-30

**Authors:** Alexane F. Rodrigue, Bruna B. Pereira, Giorgio Freije, Allison Sweet, Laili Mahmoudian, Mahmoud Aly, Salma Mahmoodianfard, Lalit Kishore, Marie-Claude Audet, Marcos F. Minicucci, K. Peter Pauls, Krista A. Power

**Affiliations:** 1 Department of Biochemistry, Microbiology and Immunology, Faculty of Medicine, University of Ottawa, Ottawa, ON K1H 8M5, Canada; arodr038@uottawa.ca; 2 School of Nutrition Sciences, Faculty of Health Sciences, University of Ottawa, Ottawa, ON K1N 6N5, Canadamaly002@plattsburgh.edu (M.A.); maudet3@uottawa.ca (M.-C.A.); 3 Internal Medicine Department, Botucatu Medical School, São Paulo State University (UNESP), Botucatu 18618-687, Brazil; 4 Department of Biological Sciences, The State University of New York Plattsburgh, Plattsburgh, NY 12901, USA; 5 School of Human Kinetics, Faculty of Health Science, University of Ottawa, Ottawa, ON K1N 6N5, Canada; 6 Department of Cellular and Molecular Medicine, Faculty of Medicine, University of Ottawa, Ottawa, ON K1H 8M5, Canada; 7 Department of Plant Agriculture, University of Guelph, Guelph, ON N1G 2W1, Canada; ppauls@uoguelph.ca

**Keywords:** gut microbiota, short-chain fatty acids, kidney beans, high-fat diet, inflammation, metabolic health, neuroinflammation, obesity

## Abstract

**Background:** Obesity-associated inflammation arises from adipose dysfunction and intestinal disturbances, including altered microbiota and short-chain fatty acid (SCFA) metabolism. Beans (*Phaseolus vulgaris*) are rich in non-digestible carbohydrates and polyphenols, but whether kidney bean varieties differing in seed coat colour exert distinct effects on inflammation in obesity remains unclear. **Objective:** To determine whether supplementation of an obesogenic high-fat (HF) diet with white or dark red kidney beans modulates gut microbiota, SCFAs, and intestinal, systemic, and neuroinflammatory outcomes. **Methods:** Male C57Bl/6N mice (*n* = 12/group) were fed a basal diet (BD; modified AIN-93G), an HF diet (60% kcal from fat), or an HF diet supplemented with 15% cooked white (HF + WK) or dark red kidney beans (HF + DK) for nine weeks. Outcomes included cecal microbiota composition, predicted KEGG pathways with taxon contributors mapped with BURRITO (a tool for linking predicted microbial functions to contributing taxa), and SCFA-related pathways; cecal and fecal SCFA concentrations; colon histomorphometry and expression of gut barrier junction and inflammatory genes; serum cytokines and adipose hormones; and hippocampal inflammatory and barrier genes. **Results:** Mice consuming bean-supplemented HF diets had higher microbial diversity, enrichment of SCFA-producing taxa (*Prevotella*, *Lactobacillus*, *Muribaculaceae*), and lower obesity-associated genera versus HF alone (*Mucispirillum*, *rc4-4*). Bean diets elevated cecal acetate and butyrate concentrations, which aligned with increases in predicted acetate kinase in both bean groups versus HF and BD, and butyrate kinase in HF + DK versus BD. Bean supplementation attenuated HF-induced reduction of goblet cells and systemic interleukin (IL)-10. The HF + DK group had lower colonic tumour necrosis factor (TNF)-α and partially attenuated hippocampal IL-6. SCFAs were inversely associated with systemic and neuroinflammatory markers in HF + DK mice. **Conclusions:** Kidney bean supplementation mitigated HF diet-induced intestinal, systemic, and neuroinflammatory disturbances in male mice, with microbiota and SCFA modulation. Further, dark red beans exerted stronger anti-inflammatory effects, highlighting the role of seed coat colour in bean-mediated obesity outcomes.

## 1. Introduction

Obesity is a multifactorial disease that presents a significant public health challenge due to its rising prevalence and its association with metabolic complications, including insulin resistance and type 2 diabetes [1]. Adipose tissue dysfunction, defined by immune cell infiltration and secretion of pro-inflammatory cytokines, promotes persistent low-grade systemic inflammation in obesity [2,3]. Additionally, the intestinal microenvironment plays a major role in inflammation associated with obesity [4,5]. Prolonged intake of high-fat diets can disrupt the gut microbiota, which can, in turn, increase intestinal permeability and immune activation [6,7,8,9]. Gut dysbiosis is characterized by reduced microbial diversity and is associated with enrichment of pro-inflammatory taxa and loss of beneficial microbes such as *Lactobacillus, Bifidobacterium*, and certain species of *Prevotella* [10,11,12,13,14,15].

Dysbiosis alters microbial fermentation patterns, shifting short-chain fatty acid (SCFA) production [16]. Acetate, propionate, and butyrate, the main SCFAs, are key microbial metabolites widely recognized for their role in gut barrier support, immune signalling, and metabolic homeostasis [17]. SCFA profiles are altered in obesity, with fecal concentrations often higher, but accompanied by shifts in composition and signalling that may impair their beneficial effects [18,19,20]. Importantly, SCFAs also influence neuroinflammation via the gut–brain axis in part by supporting blood–brain barrier integrity, limiting the entry of peripheral cytokines, and directly modulating microglial and astrocytic inflammatory signalling pathways [17,21,22]. Neuroinflammation in obesity has been linked to cognitive impairments and depressive symptoms, thus emphasizing the importance of gut-derived metabolites such as SCFAs in shaping brain health [23,24]

These findings highlight the importance of investigating the therapeutic potential of dietary interventions that target the gut microbiota composition and function. Pulses, especially common beans (*Phaseolus vulgaris*), have emerged as promising candidates due to their rich content of non-digestible carbohydrates (NDCs), which serve as substrates for beneficial microbes, including fermentable soluble fibres (e.g., pectin), oligosaccharides, and resistant starch [25,26,27,28]. Microbial fermentation of these NDCs promotes SCFA production and supports shifts in microbial composition, which may counteract dysbiosis and inflammation associated with obesity [29]. Epidemiological and clinical evidence further supports the beneficial effects of pulses on obesity-related outcomes [30]. Consumption of beans has been linked to improved satiety, regulation of appetite-related hormones [31,32,33], glucose homeostasis [34,35], lower body weight and adiposity [36], and favourable changes in lipid metabolism and blood pressure [37,38].

However, beans are compositionally diverse, and the cultivar-specific effects on gut microbiota, inflammatory outcomes, and metabolic outcomes remain poorly understood. Among bioactive components, polyphenols are particularly noteworthy for their bidirectional interactions with the gut microbiota. Most polyphenols escape absorption in the upper intestinal tract and reach the colon relatively intact, where they undergo microbial transformation into more bioactive metabolites [39]. This microbial biotransformation influences both polyphenol bioavailability and gut microbiota composition, with polyphenols modulating barrier function, immune response, and metabolic regulation [40,41,42,43,44]. Crucially, polyphenolic profiles vary widely among bean varieties, with darker-coloured beans generally exhibiting higher and more diverse phenolic content compared to lighter varieties [45,46]. Dark red kidney beans are rich in anthocyanins such as pelargonidin and cyanidin, which are largely absent in white kidney beans [47]. These compositional differences may drive cultivar-specific effects on the gut microbiota structure, SCFA production, and downstream inflammatory and metabolic outcomes. However, most studies investigating the effects of beans on obesity-related outcomes have not directly compared cultivars, and those that contrasted light and dark varieties typically used control diets containing poorly fermentable cellulose, limiting the ability to distinguish bean-specific mechanisms from the general benefits of fermentable fibre [45,48,49,50,51,52,53].

The present study addresses this gap by comparing the effects of dark red kidney beans versus white kidney beans on gut microbiota composition, SCFA profiles, as well as intestinal, systemic, and neuroinflammation during high-fat diet-induced obesity development in male mice. By incorporating soluble fibre-matched controls, this experimental design enables rigorous evaluation of cultivar-driven differences independent of fibre type. In this study, it is hypothesized that dark red kidney beans would, due to their higher and more diverse phenolic compounds compared to white beans, exert more pronounced beneficial effects on gut microbiota composition and SCFA production, with improved intestinal barrier integrity and reduced markers of inflammation in the colon, adipose tissue, circulation and hippocampus in the context of obesity.

## 2. Materials and Methods

### 2.1. Preparation and Characterization of Cooked Kidney Bean Powders and Experimental Diet Formulation

Two kidney bean cultivars (*Phaseolus vulgaris*), Yeti (white kidney bean, A35/871710W), and Dynasty (dark red kidney bean, 17/076D1-D1; Gentec Inc., Twin Falls, ID, USA), were sourced from the University of Guelph Bean Breeding Program (Guelph, ON). Beans (200 g) were soaked overnight in 750 mL water at 4 °C, rinsed and brought to a boil in 1250 mL fresh water, after which the heat was reduced to a simmer until cooked. The cooked beans and remaining water (355 mL) were blended (Waring Commercial, Torrington, CT, USA; Model # WF2212114) into a puree. The puree was placed in a pan and oven-dried at 105 °C (Moffat Inc., Winston-Salem, NC, USA; Model # E32D5), stirring every 15 min for even moisture evaporation. After 2 h, the puree was spread into a thin layer on parchment paper and dried for an additional h before it was ground into a fine powder. If there was residual moisture content, the dried powders were placed back into the oven for 20 to 40 min until 5% moisture content was achieved as determined with a moisture content analyzer (Mettler Toledo, Columbus, OH, USA; Model # HE53). The dried powder was sieved and then stored at −20 °C until use. Nutritional composition, including proximate analysis and fibre content (total and insoluble), was quantified by Bureau Veritas (Mississauga, ON, Canada), with results expressed as percentage by weight (Table S1). Additionally, the total starch content was analyzed in the kidney bean powders using the Total Starch Assay kit (Megazyme, Bray, Ireland; Cat. # K-TSTA-100A) following the manufacturer’s guidelines, since total dietary carbohydrates were calculated by difference and not measured directly. These results agreed with the calculated available carbohydrate content of the bean powders (Total carbohydrate—total dietary fibre) obtained from results provided by Bureau Veritas (Table S1) and with previous findings [27,45]. Total phenolic content (TPC) was measured in the bean powders (Table S1) and experimental diets (Table 1) using the Folin–Ciocalteu method [54] with results reported in gallic acid equivalents, which confirmed higher TPC in dark red kidney bean powder compared to white kidney bean powder, and higher TPC in both bean diets compared to control diets, while no difference in TPC was found between low- and high-fat control diets (Table S1).

### 2.2. Study Design

Five-week-old male C57Bl/6N mice were obtained from Charles River Laboratories (Kingston, NY, USA) and housed individually in standard polycarbonate cages under controlled environmental conditions (23 °C, 40% relative humidity, 12 h light/dark cycle, lights on at 07:00 h). Environmental enrichment was provided in the form of nesting material. After a one-week acclimation period with unrestricted access to water and a basal diet (BD), mice were randomly assigned to one of four dietary groups (*n* = 12 per group): BD, high-fat (HF), HF supplemented with 15% white kidney bean (HF + WK), or HF supplemented with 15% dark red kidney bean (HF + DK). In the HF group, two mice did not develop the expected obesity phenotype and were excluded from all analyses (final *n* = 10), consistent with reports that a subset of C57Bl/6N mice are resistant to diet-induced obesity [55,56]. One mouse in the HF + WK group developed malocclusion during the intervention, resulting in progressive weight loss. In accordance with animal welfare guidelines, this animal was euthanized, and the group sample size was therefore reduced to *n* = 11 for all downstream analyses. Diets were formulated by Envigo/Teklad (Madison, WI, USA) and provided *ad libitum* for nine weeks (Table 1). The BD diet was modified from the AIN-93G formulation and the HF diet was modified from the 60% kcal from fat diet (TD.160479) to include 7% total fibre (5% cellulose and 2% pectin [Gojira Fine Chemicals, LLC, Bedford Heights, OH, USA; Cat. # PE1006]) to compensate for the lack of a fermentable fibre in the original AIN-93G formulation. All experimental procedures were reviewed and approved by the Animal Care Committee at the University of Ottawa, in accordance with institutional and national guidelines for the ethical treatment of animals (Protocol #: HS-2857). Body weight and food intake were monitored twice weekly throughout the study but were reported once weekly for visualization. Whole-body composition, including lean and fat mass, was assessed at weeks 2 and 6 using Echo-MRI nuclear magnetic resonance technology (Echo-MRI™-700; EchoMRI LLC, Houston, TX, USA). Blood samples were collected from fasted mice (6 h fast) at week 7 via tail snips, allowed to clot, and serum was separated and stored at −20 °C for one week, then transferred to −80 °C for long-term storage. Fresh fecal samples were collected at week 7, while cecal contents were harvested at sacrifice, snap-frozen in liquid nitrogen, and stored at −80 °C. Mice were euthanized by decapitation at week 9; endpoint serum and tissues, including the colon, cecum (and its contents), liver, fat pads (epididymal, perinephric, and interscapular brown adipose tissue), and whole brain, were collected. One-centimetre segments from the proximal colon were snap-frozen for subsequent mRNA analysis. Liver and adipose tissues were excised, weighed, and snap frozen for biochemical and gene expression analysis. The dorsal hippocampus was dissected from a coronal brain section based on the Franklin and Paxinos mouse atlas [57], snap-frozen, and stored at −80 °C until mRNA analysis. This region was selected because obesity is associated with impairments in hippocampal-dependent cognition and memory, along with hippocampal atrophy [58,59]. Additionally, neuroinflammation within the hippocampus potentially contributes to cognitive deficits and depressive-like symptoms frequently comorbid with obesity, making it a relevant region for assessing diet-induced inflammatory responses [23,24,60].

### 2.3. 16S rRNA Library Prep and Microbiome Gene Sequencing

Cecal DNA was extracted using the QIAamp^®^ Fast DNA Stool Mini Kit (Qiagen, Hilden, Germany; Cat. # 51604), following the manufacturer’s protocol. DNA concentration was quantified by Qubit Fluorometer (Thermo Fisher Scientific, Waltham, MA, USA; Cat. # Q32850 and Q33327). The V3-V4 regions of the 16s rRNA gene were amplified as described in Illumina’s standard protocol [61]. Amplicons were pooled, spiked with 15% PhiX control DNA (Illumina, San Diego, CA, USA; Cat. # FC-110-3001), and loaded into the MiSeq reagent kit v3 (600-cycle cartridge, Illumina; Cat. # MS-102-3003) for sequencing on the Illumina MiSeq System (Cat. # SY-410-1003).

#### Bioinformatics Analysis

FASTQ files containing 300-basepair paired-end reads were demultiplexed and processed using QIIME2 (Version 2022.2) [62,63]. Denoising and chimera filtering were performed with the DADA2 plugin [64]. Amplicon sequence variants (ASVs) were aligned using MAFFT (via q2-alignment) [65], and phylogenetic trees were constructed using FastTree2 via the q2-phylogeny pipeline [66]. Taxonomic classification of ASVs was conducted using a naïve Bayes classifier (sklearn) trained on the GreenGenes database (version 13_8, 99% OTUs) (via q2-feature-classifier) [67,68,69]. The feature table was filtered to exclude unassigned phyla and low-abundance features (defined as those with fewer than 1000 reads present in fewer than five samples).

Alpha diversity metrics, including observed features and Faith’s phylogenetic diversity [70], were calculated using the QIIME2 core metrics pipeline at a rarefaction depth of 20,620 sequences per sample. Beta diversity was assessed using both weight and unweighted UNIFRAC distance matrices [71], followed by principal coordinate analysis (PCoA) [72] to visualize group clustering. Group differences in alpha diversity were evaluated using the Kruskal–Wallis test, followed by Holm–Šídák’s post hoc test [73]. Beta diversity metrics were analyzed using PERMANOVA [74] to test for significant differences in community composition across groups. Relative abundance of taxa at the phylum, genus, and species levels was compared using Mann–Whitney U tests [75], with individual ranks computed for each comparison. Multiple testing correction was applied using the Benjamini, Krieger, and Yekutieli two-stage linear step-up procedure (FDR threshold α = 0.1) [76].

Functional profiling of microbial communities was conducted using PICRUSt2 (v2.4.1) (Phylogenetic Investigation of Communities by Reconstruction of Unobserved States) [77], which inferred metagenomic content from ASV data generated in QIIME2 [62]. ASVs were aligned to reference sequences and placed into a phylogenetic tree (via GAPPA [78] using HMMER [79] and SEPP [80]. Gene family abundance was predicted via hidden-state modelling with Castor [81]. Pathway abundances were inferred (via MinPath [82]) to produce the predicted sample pathway abundances (Kyoto Encyclopedia of Genes and Genomes [KEGG] Orthology) [83]. Pathway descriptions were added using a modified R script based on the PICRUSt1 [84] categorize_by_function.py script using R [85] in RStudio software (Version 2022.7.1.554; Posit PBC, Boston, MA, USA), and they were mapped using a manually compiled mapping file retrieved from the KEGG database (https://www.genome.jp/kegg-bin/get_htext?ko00001.keg) (accessed on 17 June 2022). To assess which taxa contributed to predicted microbial functions, we reconstructed a BURRITO-style [86] analysis manually in R. Taxon-specific functional contributions from the stratified PICRUSt2 output were normalized to total function abundance and expressed as percent contribution. To visualize group-specific differences, significant taxon-function pairs (q < 0.05) were plotted using a customized ggplot2 [87] heatmap. These values were merged with sample metadata and analyzed using Kruskal–Wallis tests, with Benjamini–Hochberg correction. To assess the microbial potential for SCFA biosynthesis, a targeted list of enzyme commission (EC) numbers associated with acetate, butyrate, and propionate production was curated [88,89]. These included enzymes such as butyrate kinase (EC 2.3.1.9), acetate kinase (EC 2.7.2.1), and propionate kinase (EC 2.7.2.15), among others, which were identified using KEGG pathway maps for butanoate (ko00650) [90], pyruvate (ko00620) [91], and propanoate metabolism (ko00640) [92]. MetaCyc pathway predictions [93] were generated by PICRUSt2 based on regrouping predicted EC numbers into MetaCyc reactions and pathways. Pathways were filtered to those associated with “Fermentation to SCFAs”, as defined by MetaCyc ontology and represented in the PICRUSt2 output. Differences in KEGG pathways and targeted Metacyc pathways and corresponding ECs were statistically analyzed by Welch’s *t*-test with Benjamin–Hochberg post hoc test and visualized using STAMP software (Version 2.1.3) [94].

### 2.4. SCFAs and Branched-Chain Fatty Acids

SCFAs, including acetic, butyric, propionic, and valeric acid, and branched-chain fatty acids (BCFAs), specifically isobutyric and isovaleric acid, were quantified from cecal and fecal samples using gas chromatography (GC). Approximately 50 mg of each sample was weighed and freeze-dried for 24 h (Labconco, Kansas City, MO, USA; FreeZone 12 liter bulk tray dryer). Samples were reweighed to determine moisture content using the formula: Moisture content = [(Wet weight − Dry weight)/Wet weight]*100. Dried fecal samples were rehydrated in MilliQ water to create a 10% (*w*/*v*) solution based on dry weight, while dried cecal samples were prepared at 20% (*w*/*v*). The pH of each solution was measured using a Thermo Scientific™ Orion Star™ A111 pH metre (Thermo Fisher Scientific, Waltham, MA, USA) equipped with a ROSS MICRO pH electrode (Thermo Fisher Scientific, Waltham, MA, USA). Samples were then spiked with 10 μL of an internal standard solution containing 5.5 mmol/L 2-ethylbutyric acid (Sigma-Aldrich, St. Louis, MO, USA; Cat. # 109959-100ML) in 100% formic acid (Sigma-Aldrich, St. Louis, MO, USA; Cat. # 33015-1L). Final sample pH was adjusted to 3.5. Samples were placed into GC tubes containing glass insets (Shimadzu, Kyoto, Japan; Cat. # SZ-220-91521-03). A 1 μL aliquot of supernatant was injected into the gas chromatograph (Shimadzu, Kyoto, Japan; Nexis GC-2030), which was fitted with a 0.25 μm column (Restek, Bellefonte, PA, USA; Cat. # RSK-10226). The column flow rate was maintained at 1.5 mL/min, with a 20:1 split ratio, using helium as the carrier gas. The oven temperature was initially set to 100 °C and ramped up to 200 °C at a rate of 10 °C/min. The injector and detector temperatures were set at 200 °C and 300 °C, respectively. Each sample was analyzed in triplicate, with a total run time of 20 min per sample. SCFA concentrations were determined by comparing retention time peaks to those of Volatile Free Fatty Acid Mix (Sigma-Aldrich, St. Louis, MO, USA; Cat. # 46975-U), which was serially diluted in distilled H20 to generate a standard curve with concentrations of 0.1 mM, 0.25 mM, 0.5 mM, 1 mM, 5 mM, and 10 mM. Final SCFA and BCFA concentrations are reported as μmol/g of dry fecal or cecal weight.

### 2.5. Colon and Hippocampus mRNA Expression

Total RNA was isolated from proximal colon and dorsal hippocampus using RNA/Protein Purification Plus Kit (Norgen Biotek, Thorold, ON, Canada; Cat. #48200). For each sample, 1 μg of RNA was reverse-transcribed into complementary DNA (cDNA) using the High-Capacity cDNA Reverse Transcription Kit (Applied Biosystems, Waltham, MA, USA; Cat. # 4368814). cDNA synthesis was carried out using a T100™ Thermal Cycler (Bio-Rad, Hercules, CA, USA; Model # 1861096). Gene expression was quantified via real-time PCR using the CFX96 Touch™ (Bio-Rad, Hercules, CA, USA; Model # CFX96™ Real-Time System). Reactions were prepared with SYBR Green (Applied Biosystems, Waltham, MA, USA; Cat. #A25742), 10 mM Primers (Integrated DNA Technologies, Coralville, IA, USA), and RNAse-free H_2_O (Invitrogen, Waltham, MA, USA; Cat. # 10977015). Relative mRNA levels were calculated using the 2^−ΔΔCT^ method [95] and normalized to the geometric mean of reference genes RPLP0 and EEF2 [96], which were selected based on prior validation. Primer sequences used for target genes are listed in Table S2.

### 2.6. Colon Histomorphometry

Proximal colon cross-sections (4 μm thick), preserved in formalin, were stained with hematoxylin and eosin (H&E) or Alcian blue/nuclear fast red (AB) at the Louise Pelletier Histology Core Facility at the University of Ottawa. These sections were used to evaluate parameters related to barrier structure and function. Crypt length (from H&E-stained section), goblet cell number, and colon mucus content (from AB-stained sections) were quantified using NIH ImageJ software (Version 1.54g; U.S. National Institutes of Health, Bethesda, MD, USA) [97]. For crypt and goblet cell analysis, approximately 20 fully elongated, intact crypts per section were evaluated across 10 to 12 mice/group. Images of AB-stained sections were captured at 200× magnification in RGB JPEG format. The red channel was isolated, and an upper threshold of 160 was applied to identify the AB-stained mucus. Only mucus within the mucosal layer was analyzed, excluding luminal mucus. Stained areas were normalized to the total mucosal area and expressed as mucus content per μm^2^. All imaging was performed using a Zeiss AXIO Imager M2 (Zeiss, Oberkochen, Germany) equipped with an Axiocam 506 mono camera (Zeiss, Oberkochen, Germany).

### 2.7. Serum and Adipose Tissue Biomarkers of Metabolic Dysfunction and Inflammation

Epididymal adipose tissue (EAT) was homogenized in RIPA lysis buffer (Cell Signaling Technology, Danvers, MA, USA; Cat. # 9806) supplemented with protease inhibitors (Cell Signaling Technology; Cat. # 5871) and PMSF (Cell Signaling Technology; Cat. # 8553) using a Bead Mill 24 Homogenizer (Fisherbrand™, Thermo Fisher Scientific, Waltham, MA, USA; Cat. # 15-340-163). After centrifugation, the supernatant was carefully transferred using a syringe and needle to minimize lipid contamination. This step was repeated three times. Protein concentration was determined via BCA assay (Pierce™, Thermo Fisher Scientific, Waltham, MA, USA; Cat. # 23225), and samples were diluted 1:20. Extracted protein from EAT was diluted in 1× RIPA buffer to a final concentration of 1 mg/mL.

Circulating levels of ghrelin, gastric inhibitory polypeptide (GIP), insulin, leptin, plasminogen activator inhibitor-1 (PAI-1), and resistin were quantified in fasting serum collected at week 7 and EAT protein using a multiplex magnetic bead-based immunoassay (Bio-Rad, Hercules, CA, USA; Bio-Plex Pro™ Diabetes Panel 8-Plex, Cat. # 171-F7001M). Plates were analyzed using the Magpix system (Bio-Rad, Hercules, CA, USA; Bio-Plex^®^ MAGPIX™ Multiplex Reader, Cat. # 171015001), and data acquisition was performed with Milliplex Analyst software (Version 5.1.0.0; VigeneTech Inc., Carlisle, MA, USA#). Inflammatory markers were assessed in non-fasted endpoint serum using a Bio-Plex Pro Mouse Cytokine 23-plex Assay (Bio-Rad, Hercules, CA, USA; Cat. #M60009RDPD). The percentage of samples below the lower limit of detection for each analyte is presented in Table S6. Analytes with >20% of samples below detection were excluded from statistical analysis and not reported. Concentrations of hormones and cytokines were interpolated from standard curves generated using a five-parameter logistic regression model and were reported in pg/mL. Mouse lipopolysaccharide binding protein (LBP) enzyme-linked immunosorbent assay (ELISA) kits (HyCultBiotech, Uden, Netherlands; Cat. # HK205-01) were used to measure serum LBP concentrations as a biomarker of systemic lipopolysaccharide (LPS) exposure. The assay was performed on duplicate serum samples collected at euthanasia, as per the manufacturers’ instructions.

### 2.8. Statistical Analysis

Statistical analyses were performed using GraphPad Prism (version 10.3.1), unless otherwise specified. Two-way repeated measures ANOVA was used for longitudinal data (e.g., body weight, diet intake), with time and diet group as main effects. For outcomes measured only once (endpoint measurements) or for time-specific variables not part of a repeated-measures structure, data were analyzed by one-way ANOVA. Outliers were identified using the ROUT method (q = 1%). Normality and variance were assessed using the D’Agostino–Pearson omnibus test and Brown–Forsythe tests, respectively. Non-normally distributed data were log-transformed prior to parametric testing or analyzed using the Kruskal–Wallis test when normality could not be achieved. Significant ANOVA results were followed by Holm–Šídák’s post hoc test, while significant Kruskal–Wallis results were followed by Dunn’s post hoc test. Spearman correlation [98] was used to assess relationships between microbiota function (e.g., SCFAs) and systemic health markers (e.g., inflammation, hippocampal gene expression).

## 3. Results

### 3.1. Consumption of High-Fat Diet Influenced Body Weight and Body Composition but Not Caloric Intake

Percent body weight (BW) change was calculated relative to baseline (week 0). As depicted in Figure S1A, a statistically significant effect of time on percent BW change was observed (F (1.620, 66.41) = 861.7, *p* < 0.0001), accompanied by a significant effect of dietary group (F (3, 41) = 13.46, *p* < 0.0001). Starting at week 4, all mice receiving the HF diet exhibited a significantly elevated weight gain relative to BD, with this difference remaining significant for the remainder of the study (see Table S3 for exact *p* values). In mice consuming bean-supplemented HF diets, increased weight gain, compared to BD, started at week 1, which is a trend that persisted throughout the experimental timeline (Figure S1A, Table S3). There were no significant differences in % BW change between HF, HF + WK, and HF + DK at any point throughout the study. At the study endpoint, final BW was significantly elevated in HF, HF + WK and HF + DK groups compared to BD (Figure S1B). Analysis of dietary intake as kcal/day, calculated by multiplying average daily food consumption (g/day) by the energy density of the assigned diet (kcal/g), as provided in Table 1, revealed a significant effect of time (F (6.872, 281.8) = 10.72, *p* < 0.0001), but no effect of diet group (Figure S1C). When intake was expressed in grams/day, both time (F (4.134, 163.8) = 16.40, *p* < 0.0001) and diet (F (3, 40) = 71.57, *p* < 0.0001) significantly influenced consumption (Figure S1D). BD mice consistently consumed more grams of diet per day than all HF-based groups across the study, but because HF diets were more energy dense, these gram differences did not result in differences in total caloric intake.

Body composition was evaluated via EchoMRI at weeks 2 and 6. Lean mass increased significantly over time (F (1, 41) = 204.7, *p* < 0.0001) and showed a significant time x diet interaction (F (3, 41) = 5.220, *p* = 0.003), although the overall main effect of diet was not significant. Bean-fed mice exhibited modestly higher lean mass (~1 g) compared with HF at both timepoints, although these differences did not reach statistical significance (Figure S1E). Fat mass increased over time (F (1, 41) = 535.6, *p* < 0.0001) and was affected by diet (F (3, 41) = 8.780, *p* = 0.0001), with a significant time × diet interaction (F (3, 41) = 11.47, *p* < 0.0001) (Figure S1F). At week 6, mice fed HF, HF + WK, and HF + DK diets exhibited significantly increased fat mass relative to the BD group (*p* < 0.0001) (Figure S1G). Further analysis of adipose tissue depots revealed significantly greater relative epididymal and perirenal fat pad weights in all HF-fed groups compared to the BD group (*p* < 0.0001). No significant differences were observed in relative brown adipose tissue weight or liver weights across dietary groups (Table S4).

### 3.2. Consumption of Bean-Supplemented High-Fat Diets Altered the Cecal Microbiota Composition and Function in Male Mice

#### 3.2.1. Microbial Community Diversity and Structure

**Figure 1:**
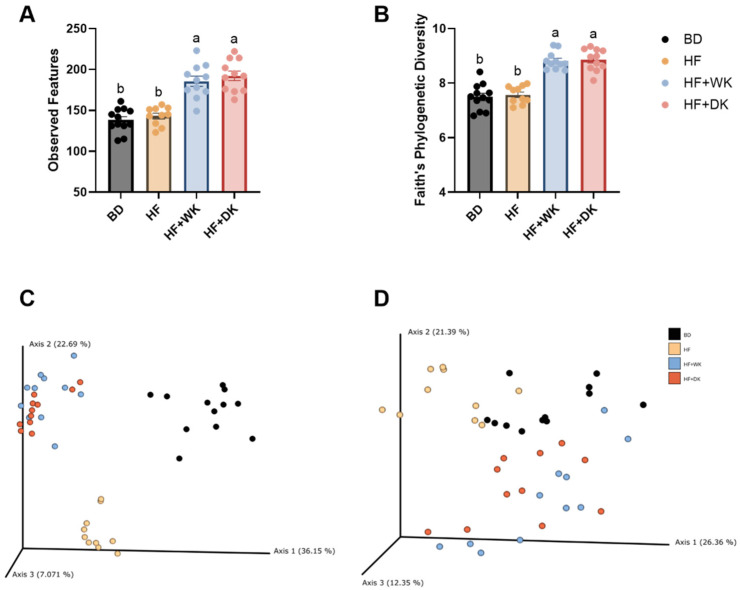
Diet alters the taxonomic diversity of the cecal microbiota. Cecal microbiota α-diversity was assessed using (**A**) observed features; (**B**) Faith’s phylogenetic diversity. β-diversity was visualized by principal coordinates analysis (PCoA) of (**C**) unweighted and (**D**) weighted UniFrac distance matrices, demonstrating clustering by dietary group. Each point represents one mouse, and diet groups are denoted by colour (BD: black; HF: orange; HF + WK: blue; HF + DK: red). Groups not sharing a lowercase letter are significantly different (*p* < 0.05). BD = Basal diet, HF = High-fat diet, HF + WK = HF + white kidney bean, HF + DK = HF + dark red kidney bean.

To establish the baseline effects of diet on cecal microbial diversity, we first compared the HF group to the BD group. As shown in Figure 1A,B, HF feeding did not significantly alter α-diversity relative to BD, as indicated by both observed features and Faith’s phylogenetic diversity (PD).

In contrast, as shown in Figure 1C,D, β-diversity analyses (unweighted and weighted UNIFRAC) showed clear separation between BD and HF groups, confirming that the high-fat diet shifted overall community structure (*p* < 0.001, post hoc q = 0.0012).

We next evaluated whether kidney bean supplementation modified these HF-induced changes. Both HF + WK and HF + DK groups exhibited higher α-diversity than HF, with significant increases in observed features and Faith’s PD (*p* < 0.0001) (Figure 1A,B). These effects were consistent across both bean types, and no differences were detected between HF + WK and HF + DK. Therefore, bean supplementation increased microbial richness and altered phylogenetic diversity compared to HF feeding alone.

β-diversity analyses further supported these findings. PCoA plots (Figure 1C,D) demonstrated that both bean-supplemented groups clustered distinctly from HF (*p* < 0.001), indicating that white and dark red kidney bean supplementation shifted the overall microbial community structure away from the HF profile. Clustering patterns also showed partial overlap between HF + WK and HF + DK, with no significant differences in β-diversity between the bean groups, suggesting similar community-level effects of the two cultivars.

**Figure 2:**
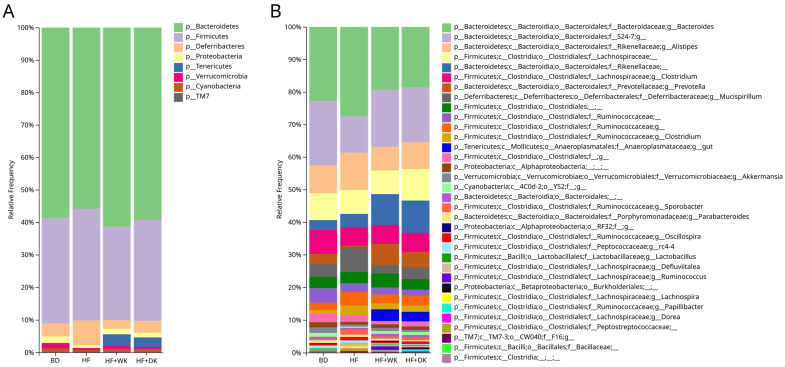
Cecal microbiota composition is modified by kidney bean supplementation. Relative abundance of bacterial taxa at the (**A**) phylum and (**B**) genus levels in cecal samples. Taxa representing >0.03% of total sequences in at least one sample are shown. Relative abundances were calculated from 16S rRNA gene sequencing data and are expressed as the proportion of total sequences per sample. BD = Basal diet; HF = High-fat diet; HF + WK = HF + white kidney bean; HF + DK = HF + dark red kidney bean.

Cecal microbial composition (Table 2 and Figure 2) showed distinct shifts across groups, with group differences summarized in Table 2. To first define the effects of high-fat feeding, we compared HF with BD. At the phylum level, HF feeding increased the relative abundance of *Deferribacteres* and *TM7*, while reducing *Proteobacteria*, *Verrucomicrobia*, and *Cyanobacteria*, indicating a shift toward a dysbiotic profile commonly associated with high-fat diets.

We next assessed whether kidney bean supplementation modified HF-induced phylum-level changes. Relative to HF, both HF + WK and HF + DK groups showed reductions in *Deferribacteres* and increases in *Tenericutes*, indicating partial reversal of specific HF-associated alterations. Additionally, only the HF + WK group exhibited decreased *Firmicutes* and increased abundance of *Bacteroidetes* and *Proteobacteria* compared to the HF group. Together, these patterns suggest that bean supplementation alters HF-driven shifts in phylum-level composition, while also introducing modest cultivar-specific differences in how individual phyla respond.

To further characterize these HF-induced disruptions and bean-mediated corrections, we next examined genus-level changes. At the genus level, HF feeding increased *Clostridia o.*, *Lachnospira*, *Clostridium* (*Rumminococcaceae* family), *Oscillospira*, *Sporobacter*, *Mucispirillum*, and *F16 g.*, and decreased *Prevotella*, *S24-7* (*Muribaculaceae*), *Burkholderiales*, *Akkermansia*, and *YS2* compared to BD. These shifts are consistent with high-fat diet-induced dysbiosis characterized by reduced beneficial taxa and expansion of genera associated with metabolic dysfunction [10,11,12,13,14,15].

Relative to the HF group, both HF + WK and HF + DK increased *Lactobacillus*, *Ruminococcus*, *Oscillospira*, *Prevotella*, *Rikenellaceae g.*, *S24-7* (*Muribaculaceae*), *Burkholderiales*, and *Anaeroplasmataceae gut* sp., and decreased abundance of *Defluviitalea*, *Lachnospira*, *rc-4-4*, *Bacteroides*, and *Mucispirillum*. Notably, *Anaeroplasmatacaea gut* sp. were absent in both BD and HF groups, but appeared exclusively in the bean-supplemented groups, highlighting a bean-specific enrichment. Additionally, HF + WK mice showed decreased *Clostridia o.*, *Clostridiales f.*, *Clostridium* (*Rumminococcaceae* family), *Oscillospira*, and *Alistipes*, whereas the HF + DK group had increased *Dorea* and decreased *Parabacteroides* compared to HF. No significant differences were observed between HF + WK and HF + DK groups (Figure S2 and Table S5), indicating that the two bean cultivars shifted the community in similar directions with modest genus-level distinctions. Many of the taxa restored by bean supplementation, including *Lactobacillus*, *Prevotella*, and *S24-7*, are associated with SCFA production and gut barrier support, suggesting that these microbial shifts may contribute to the improved inflammatory outcomes observed in bean-fed mice.

At the species level (Table S6), several taxa contributed to the observed genus-level changes. The reduction in *Alistipes* and *Clostridium* detected in HF + WK was primarily driven by significant decreases in *Alistipes finegoldii*, *Alistipes massiliensis*, and *Clostridium Methylpentosum*. While bean-driven increases in *Rumminococcus*, *Lactobacillus*, *Dorea* (HF + DK only), and *Papillibacter* were attributable to the higher abundance of *Rumminococcus lactaris*, *Lactobacillus hamsteri*, *Dorea longicatena*, and *Papillibacter cinnamivorans*.

#### 3.2.2. Predicted Function of the Microbiota

**Figure 3:**
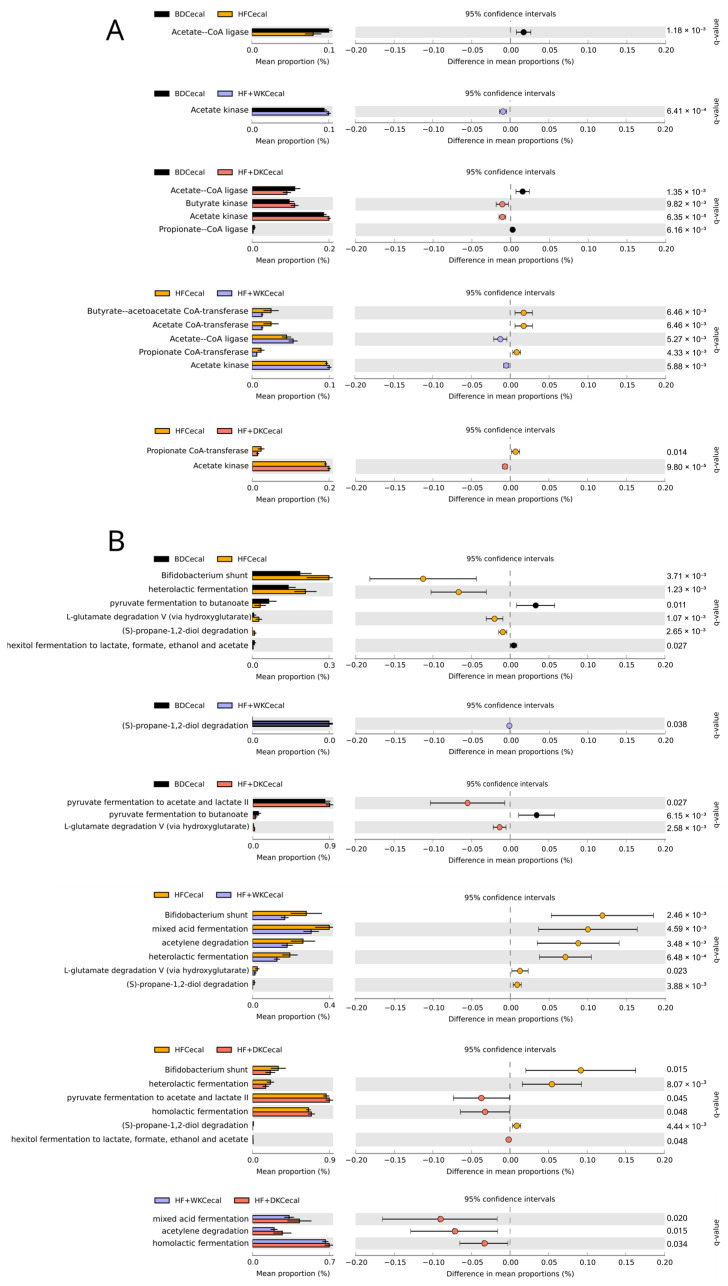
Differential abundance of microbial enzymes and pathways involved in short-chain fatty acid metabolism. STAMP analysis of PICRUSt2 predicted enzyme pathways revealed significant differences in the relative abundance of key (**A**) microbial enzyme commission numbers (EC) and (**B**) MetaCyc pathways involved in SCFA production across dietary groups. BD = Basal diet, HF = High-fat diet, HF + WK = HF + white kidney bean, HF + DK = HF + dark red kidney bean.

Functional predictions of the cecal microbiota were generated using PICRUSt2, and differences in enzyme commission numbers (EC) and KEGG pathway abundance between groups were analyzed using STAMP [94]. Functional predictions revealed diet-dependent shifts in SCFA metabolism, such that both bean-supplemented groups showed significant increases in acetate kinase compared to HF (HF + WK: q = 5.88 × 10^−3^; HF + DK: q = 9.80 × 10^−5^) and BD (HF + WK: q = 6.41 × 10^−4^; HF + DK: q = 6.35 × 10^−5^), indicating elevated potential for acetate biosynthesis (Figure 3A). Butyrate kinase was significantly elevated only in HF + DK compared to BD (q = 9.82 × 10^−3^). Propionate CoA-transferase levels did not differ significantly between BD and bean groups but were significantly lower in bean groups compared to the HF group (HF + WK: q = 4.33 × 10^−3^; HF + DK: q = 0.014).

Metacyc analysis showed that, relative to BD, HF feeding was associated with enrichment of mixed and amino acid-associated fermentation pathways, and reduced representation of butyrate-producing routes (Figure 3B). Conversely, BD exhibited higher abundance of the pathways pyruvate fermentation to butanoate and hexitol fermentation to lactate, formate, ethanol and acetate, which contribute to butyrate and acetate production, indicating that high-fat feeding alters microbial fermentation pathway profiles relative to BD. Comparison of HF and HF + WK revealed enrichment of multiple fermentation pathways in HF, with no pathways enriched in HF + WK. Pathways elevated in HF included mixed acid fermentation (q = 4.59 × 10^−3^), heterolactic fermentation (q = 6.48 × 10^−4^), and amino acid–associated degradation routes (L-glutamate degradation V, q = 0.023), suggesting that white kidney beans did not substantially alter the HF-associated fermentation profile. Pathways enriched in HF + DK compared to HF included pyruvate fermentation to acetate and lactate (q = 0.045), homolactic fermentation (q = 0.048), and hexitol fermentation to lactate, formate, ethanol and acetate (q = 0.048). Comparing HF + DK and HF + WK revealed a higher abundance of fermentation-related pathways in HF + DK, including mixed acid fermentation (q = 0.02), homolactic fermentation (q = 0.034), and acetylene degradation (q = 0.015). Together, these findings indicate that although both bean-supplemented diets increased predicted acetate biosynthetic capacity, only dark red kidney bean supplementation was associated with a coordinated shift in fermentative pathways toward acetate-generating metabolism under high-fat conditions.

**Figure 4:**
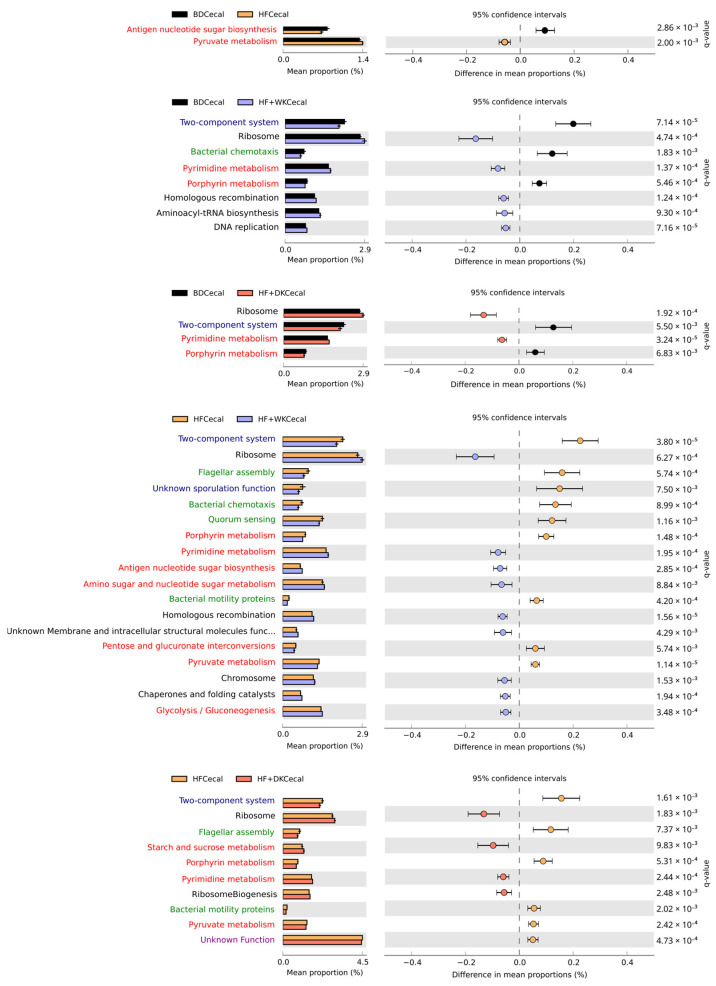
Differentially abundant microbial pathways. STAMP analysis of PICRUSt2 predicted KEGG pathways revealed significant differences in microbial functional potential across diets. Pathways are colour-coded by Brite hierarchy: Red = Metabolism, Blue = Environmental Information Processing, Black = Genetic Information Processing, Green = Cellular Process, Purple = Poorly Characterized. BD = Basal diet, HF = High-fat diet, HF + WK = HF + white kidney bean, HF + DK = HF + dark red kidney bean.

KEGG pathway analysis revealed broader functional changes in bean-supplemented groups compared to HF alone, which had functional profiles more closely resembling those of BD-fed mice (Figure 4). In fact, HF-fed mice had only minor functional shifts from BD, such as increased antigen nucleotide sugar biosynthesis (q = 2.86 × 10^−3^) and reduced pyruvate metabolism (q = 2.0 × 10^−3^). In contrast, compared to HF both bean diets enriched biosynthetic and genomic maintenance pathways, including starch and sucrose metabolism (HF + DK only, q = 9.83 × 10^−3^), pyrimidine metabolism (HF + WK: q = 1.95 × 10^−4^; HF + DK: q = 2.44 × 10^−4^), ribosome (HF + WK: q = 6.27 × 10^−4^; HF + DK: q = 1.83 × 10^−3^), amino sugar and nucleotide sugar metabolism (HF + WK only, q = 8.84 × 10^−3^), and reductions in environmental sensing and motility pathways such as two-component system (HF + WK: q = 3.80 × 10^−5^; HF + DK: q = 1.61 × 10^−3^), flagellar assembly (HF + WK: q = 5.74 × 10^−4^; HF + DK: q = 7.37 × 10^−3^), and pyruvate metabolism (HF + WK: q = 1.14 × 10^−5^; HF + DK: q = 2.42 × 10^−4^). No significant differences were observed between HF + WK and HF + DK, suggesting similar microbial functional effects of both bean varieties. Overall, kidney bean supplementation shifted microbial functional effects away from those observed in BD- and HF-fed mice and toward pathways supporting carbohydrate and nucleotide biosynthesis and SCFA production.

**Figure 5:**
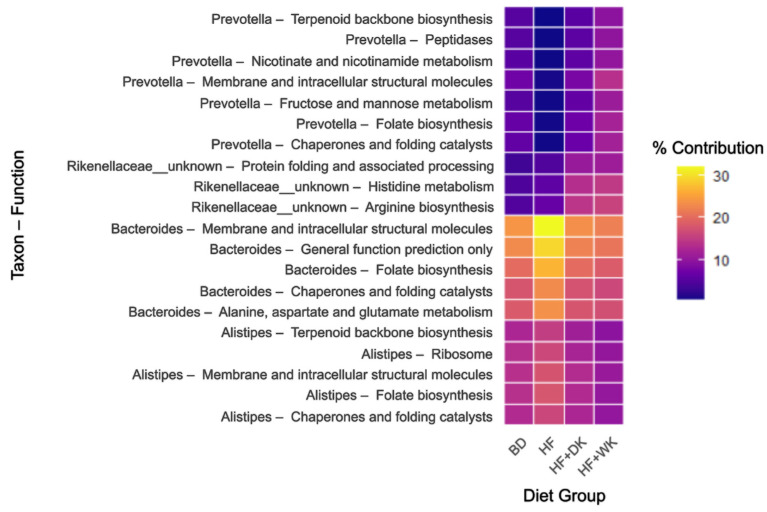
Taxon–function heatmap of top contributing microbial pathways. Heatmap of the top 20 taxon–function pairs based on maximum percent contribution across diet groups. Rows represent the highest-contributing taxa to a given function, and columns indicate dietary groups. Colour intensity reflects the mean percent contribution of each taxon to each function within a group. BD = Basal diet, HF = High-fat diet, HF + WK = HF + white kidney bean, HF + DK = HF + dark red kidney bean.

Relationships between microbial taxa and predicted functions were assessed using BURRITO-style analysis [86], revealing diet-dependent shifts among the top 20 taxon–function pairs (Figure 5, Table S7). *Prevotella*-associated functions showed significant overall diet effects (all q < 1.46 × 10^−5^), with HF feeding significantly reducing Prevotella contributions to fructose and mannose metabolism, folate and nicotinate metabolism, and structural molecule production, compared to BD (all q < 0.006), with restoration observed in both bean-fed groups relative to HF (q < 0.002). *Rikenellaceae*-linked functions had significant diet effects (all q < 2.64 × 10^−5^) caused by changes in histidine and arginine metabolism, along with protein folding, which were elevated in bean-fed mice compared to both BD and HF (all q < 0.006). *Bacteroides*-associated pathways were significantly altered by diet (all q < 0.01), as the HF group had enriched membrane and intracellular structural molecule, and folate biosynthesis pathways relative to all other groups (all q < 0.04), and these taxa-linked functions were suppressed with bean supplementation compared to HF (all q < 0.04). *Alistipes*-associated functions followed a similar pattern, showing HF enrichment with attenuation in HF + WK group (all q < 0.02), but not in HF + DK group. Exact q-value and post hoc q-values for all taxon–function comparisons are provided in Table S7. A BURRITO view opened at KEGG Level 1 for *Prevotella* (Figure S3) illustrates the distribution of its predicted functional contributions across broad categories in each diet group.

#### 3.2.3. Short-Chain Fatty Acids

**Figure 6:**
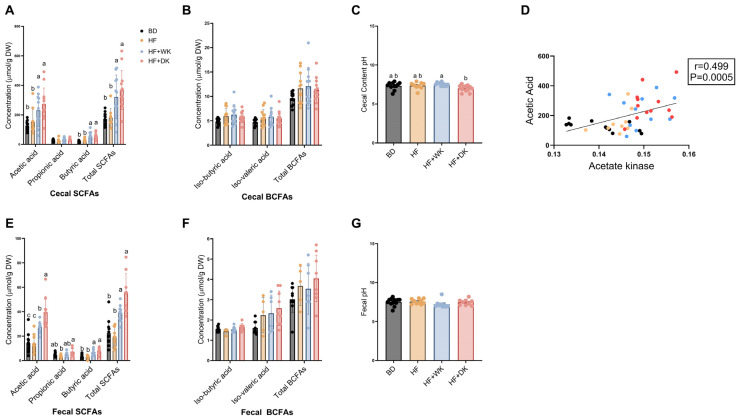
Cecal and Fecal SCFA and BCFA concentrations across diet groups. Concentrations of individual and total short-chain fatty acids (SCFAs) and branched-chain fatty acids (BCFAs) were measured from fecal content collected at week 7 and cecal contents following 9 weeks of dietary intervention. (**A**) Cecal SCFAs. (**B**) Cecal BCFAs. (**C**) Cecal content pH. (**D**) Spearman correlation between acetic acid and predicted acetate kinase abundance. (**E**) Fecal SCFAs. (**F**) Fecal BCFAs. (**G**) Fecal pH. Data are presented as mean ± SEM. Groups not sharing a lowercase letter are significantly different (*p* < 0.05). Each point represents an individual mouse and diet groups are denoted by colour (BD: black; HF: orange; HF + WK: blue; HF + DK: red). BD = Basal diet, HF = High-fat diet, HF + WK = HF + white kidney bean, HF + DK = HF + dark red kidney bean.

To assess how dietary treatments influenced microbial fermentation products, cecal SCFA and BCFA concentrations were measured across all groups (Figure 6). Total cecal SCFA concentrations showed a significant overall effect of diet (*p* < 0.0001), with post hoc analyses indicating total SCFAs were significantly elevated in HF + WK (*p* = 0.002) and HF + DK (*p* < 0.0001) diets compared to BD, and also increased relative to HF (*p* = 0.0086 for HF + WK; *p* = 0.0004 for HF + DK) with no significant differences between bean diets (*p* = 0.2973) (Figure 6A). This increase was driven in part by elevated acetic acid concentrations (*p* = 0.0001), which were significantly higher in the HF + WK (*p* = 0.0202) and HF + DK (*p* = 0.0006) groups compared to BD, and also elevated relative to HF (*p* = 0.0349 for HF + WK; *p* = 0.0018 for HF + DK), with no differences between beans. Butyric acid followed a similar pattern (*p* < 0.0001), with significantly higher levels in the HF + WK (*p* < 0.0001) and HF + DK (*p* < 0.0001) groups compared to BD, and significantly higher in HF + WK (*p* = 0.0094) and HF + DK (*p* = 0.0006) groups compared to HF, with no differences between bean groups. In contrast, there were no significant differences in propionic acid between groups. BCFAs were also measured in cecal samples collected at the study endpoint (Figure 6B). No significant differences were observed between groups for iso-butyric acid, iso-valeric acid or total BCFAs. These measured shifts in acetic and butyric acid and lack of increase in propionic acid were consistent with functional predictions of enzyme profiles in the bean-supplemented groups. Acetic acid further demonstrated a significant positive correlation with acetate kinase abundance (r = −0.499, *p* = 0.0005), an enzyme associated with acetate-producing pathways (Figure 6D). Cecal content pH was significantly reduced in the HF + DK group compared to HF + WK (*p* = 0.005) (Figure 6C).

In feces, similar bean-induced increases in SCFA concentrations were observed (Figure 6E), supported by significant overall diet effects for total SCFAs (*p* < 0.0001), acetic acid (*p* < 0.0001), butyric acid (*p* < 0.0001), and propionic acid (*p* = 0.007). For example, both bean diets increased butyric acid and total SCFAs compared to BD and HF groups (all *p* < 0.001), however, HF + WK beans induced an intermediate increase in acetic acid compared to BD and HF (*p* = 0.0056 for both comparisons), which was significantly lower than that induced by HF + DK group (*p* = 0.0085). Further, a significant increase was observed in propionic acid in the HF + DK compared to the HF group (*p* = 0.005) (Figure 6E). There were no observed differences between groups in fecal BCFAs or in fecal pH (Figure 6F,G).

### 3.3. Consumption of Bean-Supplemented Diets Improved Colon Morphology and Altered Intestinal Inflammation

**Figure 7:**
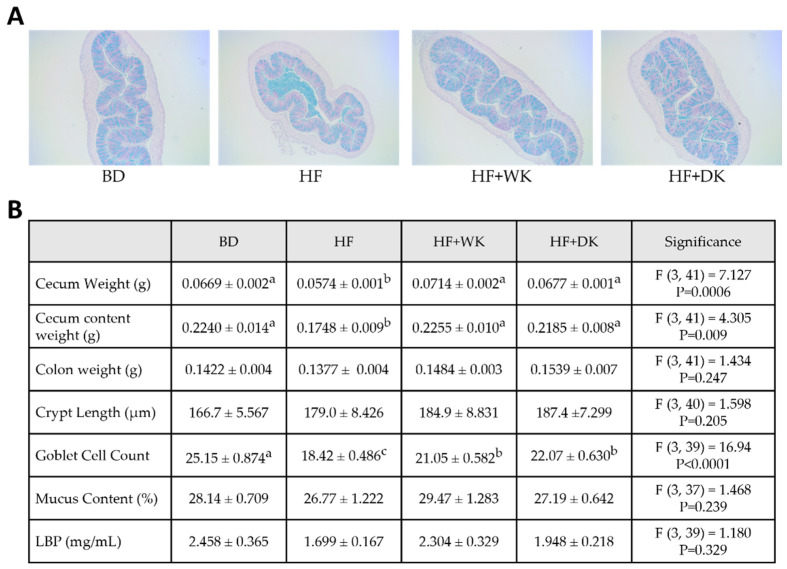
Representative colon histology and quantitative measures of intestinal morphology and barrier health. (**A**) Representative alcian blue-stained colon sections from each group (5× magnification). Images show structural differences in crypt architecture and goblet cell density. (**B**) Quantitative measures of intestinal parameters, including cecum, cecum content and colon weight, crypt length, goblet cell counts, mucus content, and serum LBP levels. Groups not sharing a lowercase letter are significantly different (*p* < 0.05). BD = Basal diet, HF = High-fat diet, HF + WK = HF + white kidney bean, HF + DK = HF + dark red kidney bean.

**Figure 8:**
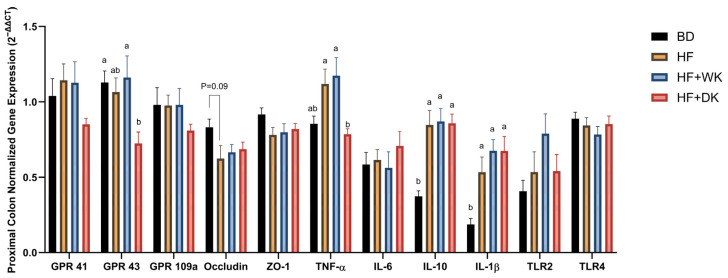
Relative gene expression in the proximal colon of mice across experimental groups. Expression levels of genes involved in short-chain fatty acid (SCFA) sensing (GPR41, GPR43, GPR109a), epithelial barrier integrity (occludin, ZO-1), pro- and anti-inflammatory signalling (TNF-α, IL-6, IL-1β, IL-10), and innate immune recognition (TLR2, TLR4) were quantified via PCR. Data are normalized to housekeeping genes and presented as mean ± SEM. Groups not sharing a lowercase letter are significantly different (*p* < 0.05). BD = Basal diet, HF = High-fat diet, HF + WK = HF + white kidney bean, HF + DK = HF + dark red kidney bean.

To evaluate how bean-enriched diets influence colon health, cecum and colon size were assessed, as well as proximal colon barrier function and integrity by histomorphometrics, serum LBP concentrations (Figure 7), and quantification of gene expression in the proximal colon (Figure 8). Overall ANOVA results are shown in Figure 7B, post hoc findings are reported here. Mice in the HF group had significantly lower cecum weights compared to those in the BD group (*p* = 0.014). However, mice consuming an HF diet supplemented with either white kidney or dark kidney beans had restored cecum weights (*p* = 0.0004 and *p* = 0.0086, respectively, vs. HF), reaching a level not significantly different from the BD group. Similarly, cecum content weight was reduced in HF-fed mice compared to BD (*p* = 0.021), and both bean-supplemented groups showed significant increases relative to HF (HF + WK: *p* = 0.02; HF + DK: *p* = 0.03), with no differences from BD. No significant differences in colon weight or length were observed across dietary groups (Figure 7B).

Consumption of all high-fat diets resulted in a significant reduction in goblet cell number compared to BD, with the HF group having the lowest goblet cell count (*p* < 0.0001). Both bean diets attenuated HF-induced loss of goblet cells, as they were significantly elevated in HF + WK and HF + DK groups compared to HF (*p* = 0.024 and *p* = 0.002, respectively) but remained significantly lower than in BD-fed mice (HF + WK: *p* = 0.0006; HF + DK: *p* = 0.006). Crypt length and mucus content remained unchanged across groups. To determine whether these morphological changes were accompanied by alterations in intestinal permeability, serum LBP levels were assessed. However, no significant differences were observed between groups (Figure 7B), suggesting limited evidence for altered intestinal permeability based on serum LBP levels.

Gene expression analysis (Figure 8) revealed no significant differences in SCFA receptors GPR41 and GPR109a; however, GPR43 expression was significantly different across groups (ANOVA *p* = 0.01). Post hoc comparisons revealed that GPR43 expression was significantly reduced in the HF + DK group compared to BD (*p* = 0.03) and HF + WK (*p* = 0.02), while the HF group did not differ significantly from any other group. For epithelial barrier function, occludin showed a trend toward statistical significance (*p* = 0.07), with highest expression in the BD group; it appeared lower in the HF group (trend *p* = 0.09), showing a possible disruption of barrier integrity in HF-fed mice. This trend was not observed in the bean-supplemented groups. ZO-1 expression did not differ between groups. Regarding inflammatory signalling, TNF-α expression was significantly altered by diet (*p* = 0.003), with reduced TNF-α in the HF + DK group compared to both HF (*p* = 0.02) and HF + WK (*p* = 0.008), indicating a potential anti-inflammatory effect with dark bean supplementation. IL-10 and IL-1β expression were significantly elevated in all high-fat-fed mice compared to BD (both *p* < 0.0001), while IL-6 expression remained unchanged. The innate immune receptors, TLR2 and TLR4, involved in microbial–host interactions, showed no significant differences between groups.

### 3.4. Bean Consumption Improved Systemic Inflammation and Metabolic Hormones

To investigate the systemic and adipose-specific effects of bean-supplemented high-fat diets, concentrations of adipose and endothelial-derived adipokines (leptin, resistin, PAI-1), gut-derived hormones (GIP and ghrelin), and markers of glucose homeostasis (fasting glucose, insulin and HOMA-IR) were assessed in fasted serum. In addition, leptin, resistin, and PAI-1 protein levels were quantified in EAT to assess local inflammatory and metabolic responses (Table 3). As shown in Table 3, overall ANOVA results indicated significant diet effects for several markers; post hoc comparisons were performed. Serum PAI-1 levels were significantly lower in the HF group compared to BD (*p* = 0.02), with no differences observed between the bean-fed groups and BD. On the other hand, PAI-1 concentrations in adipose tissue remained unchanged across groups. Leptin levels were significantly elevated in both serum and adipose tissue in the HF (*p* = 0.02 for serum; *p* = 0.05 for EAT) and HF + DK (*p* = 0.003 for serum; *p* = 0.002 for EAT) groups when compared to BD, while leptin levels in the HF + WK group were significantly increased in adipose tissue (*p* = 0.02) but not in serum when compared to BD. Although serum resistin levels were not significantly altered between groups, adipose resistin concentrations were significantly reduced in the HF + DK group compared to BD (*p* = 0.0173). Among the gut-derived hormones, serum GIP levels were significantly reduced in all high-fat-fed groups compared to BD (*p* = 0.02). Fasting serum HOMA-IR values were significantly elevated in all high-fat-fed groups compared to BD (HF *p* = 0.02; HF + WK *p* = 0.01; HF + DK *p* = 0.01), indicating impaired insulin sensitivity. Similarly, fasting blood glucose levels were significantly increased in HF (*p* = 0.003), HF + WK (*p* = 0.003), and HF + DK (*p* = 0.0005) groups relative to BD. In contrast, fasting insulin concentrations did not differ significantly between groups (although nominally higher in high-fat groups), suggesting that changes in HOMA-IR were driven primarily by elevated glucose rather than insulin hypersecretion.

Markers of inflammation were assessed in endpoint serum using a cytokine 23-plex bead-based immunoassay. Most cytokines showed no significant differences between groups; however, some notable patterns emerged (Table S8). The anti-inflammatory cytokine IL-10 was significantly altered by diet (*p* = 0.017), with reduced IL-10 in the HF group compared to BD (mean: 8.06 pg/mL vs. 25.74 pg/mL; *p* = 0.0138), indicating suppressed anti-inflammatory capacity with HF feeding. Interestingly, serum IL-10 levels following bean supplementation did not differ from HF or BD groups (HF + WK: 13.23 pg/mL; HF + DK: 18.90 pg/mL), suggesting a partial improvement in the serum anti-inflammatory profile induced by beans. There was a trend for RANTES (*p* = 0.053), which was elevated in the BD group compared to HF (BD: 32.75 pg/mL; HF: 13.81 pg/mL, *p* = 0.04). No other cytokines showed statistically significant differences between groups (Table S8).

### 3.5. Bean Consumption Modulated Hippocampal Inflammatory and Blood–Brain Barrier Gene Expression

**Figure 9:**
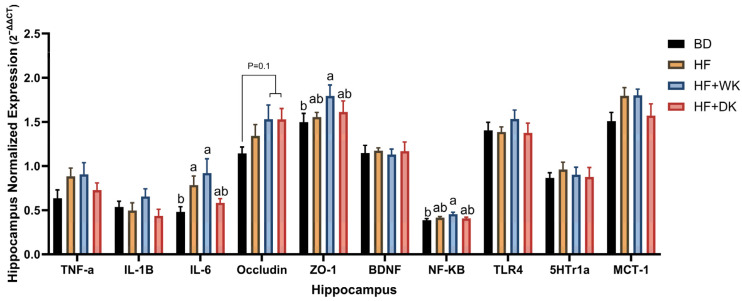
Relative gene expression in the hippocampus of mice across experimental groups. Expression levels of genes related to neuroinflammation (TNF-α, IL-1β, IL-6, NFKB, TLR4), blood–brain barrier integrity (Occludin, ZO-1), neuroplasticity and neurotransmission (BDNF, 5HT1a), and short-chain fatty acid transport (MCT-1) were quantified via PCR. Data are normalized to housekeeping genes and presented as mean ± SEM. Groups not sharing a lowercase letter are significantly different (*p* < 0.05). BD = Basal diet, HF = High-fat diet, HF + WK = HF + white kidney bean, HF + DK = HF + dark red kidney bean.

Markers of inflammation, blood–brain barrier (BBB) integrity, neuroplasticity, and SCFA transport were analyzed in the hippocampus as part of assessing diet-related effects on brain health in the context of obesity (Figure 9). Overall ANOVA results indicated significant diet effects for IL-6 (*p* = 0.008), with IL-6 expression significantly elevated in the HF (*p* = 0.05) and HF + WK (*p* = 0.009) groups compared to BD, while HF + DK levels were not significantly different from BD or HF. Nuclear factor kappa-light-chain-enhancer of activated B cells (NF-κB) expression also demonstrated a significant overall diet effect (*p* = 0.03), as NF-κB was significantly increased in HF + WK compared to BD (*p* = 0.02), with HF and HF + DK showing intermediate levels. No significant differences were observed in TNF-α, IL-1β or TLR4 between groups. Blood–brain barrier markers were modulated by diet, as ZO-1 expression was significantly different (*p* = 0.04), which was elevated in HF + WK compared to BD (*p* = 0.038), while HF and HF + DK showed intermediate levels not statistically different from either BD or HF + WK. Occludin expression showed an overall trend (*p* = 0.07), with a non-significant increase in both HF + WK and HF + DK groups compared to BD (*p* = 0.1 for both bean groups), suggesting potential enhancement of barrier integrity with bean supplementation. Genes relating to neuroplasticity (BDNF, 5HT1a) and SCFA transport (MCT-1) were not significantly different between groups.

### 3.6. Relationships Between SCFAs and Intestinal, Systemic, and Neuroinflammation

To further explore potential interactions between diet, microbiota, and inflammatory outcomes, we conducted exploratory correlations between SCFAs, key microbial taxa, and markers of colonic, systemic and brain inflammation. We focused on taxa that were significantly altered by bean supplementation and selected inflammatory markers that were significantly altered or tended to be different between groups. Serum TNF-α was included despite not being significantly altered because it has been linked to GPR43 expression [99], which was also altered in our study.

**Figure 10:**
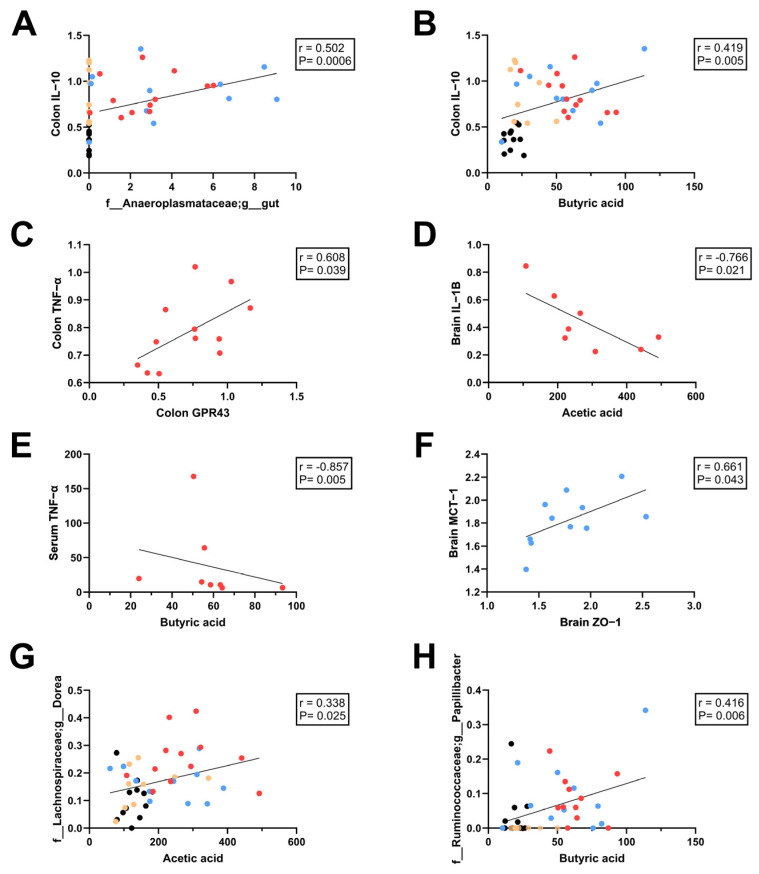
Short-chain fatty acid and microbiota-related correlations with inflammatory markers in colon, systemic, and brain tissues. (**A**) Correlation between *Anaeroplasmataceae gut* and colon IL-10 across all dietary groups. (**B**) Correlation between colon IL-10 and cecal butyric acid across all dietary groups. (**C**) Correlations between colon TNF-α and cecal butyric acid within HF + DK group. (**D**) Correlation between brain IL-1β and cecal butyric acid within HF + DK group. (**E**) Correlation between serum TNF-α and cecal butyric acid within HF + DK group. (**F**) Correlation between brain MCT-1 and brain ZO-1 within the HF + WK group. (**G**) Correlation between *Dorea* and cecal butyric acid across all dietary groups. (**H**) Correlation between *Papillibacter* and cecal butyric acid across all dietary groups. Correlations were assessed using Spearman correlation, with correlation coefficients (*r*) and *p* values shown. The solid line represents a linear fit shown for visualization purposes only. Each point represents an individual mouse and diet groups are denoted by colour (BD: black; HF: orange; HF + WK: blue; HF + DK: red). BD = basal diet, HF = High-fat diet, HF + WK = HF + white kidney bean, HF + DK = HF + dark red kidney bean.

Correlation analysis revealed significant associations between microbial and SCFA measures with inflammatory markers across tissues (Figure 10). Across all groups combined, *Anaeroplasmataceae gut* (r = 0.608, *p* = 0.039; Figure 10A) and cecal butyric acid (r = 0.419, *p* = 0.005; Figure 10B) were positively correlated to colonic IL-10. In the HF + DK-fed mice, colonic TNF-α expression was positively correlated with GPR43 (r = 0.60, *p* = 0.039; Figure 10C), while both cecal acetic acid and butyric acid were inversely correlated with systemic and neuroinflammatory markers, including serum TNF-α (r = −0.857, *p* = 0.005: Figure 10E) and hippocampal IL-1β (r = −0.766, *p* = 0.021: Figure 10D) respectively. In the HF + WK group, hippocampal ZO-1 expression positively correlated with hippocampal MCT-1 (r = 0.661, *p* = 0.043: Figure 10F), suggesting a potential link between SCFA transport and BBB integrity. Across all groups, cecal acetic acid concentrations were positively correlated with the relative abundance of *Dorea* (r = 0.338, *p* = 0.025; Figure 10G), and butyric acid concentrations were positively correlated with the relative abundance of *Papillibacter* (r = 0.416, *p* = 0.006; Figure 10H), consistent with the role of these genera in fermentative metabolism. Overall, these findings indicate that individual variability in microbial fermentation capacity within the bean groups could contribute to differences in systemic and neuroinflammatory outcomes in the context of HF feeding.

## 4. Discussion

This study investigated whether consumption of kidney beans rich in polyphenols and NDCs could mitigate high-fat diet-induced disturbances in the gut microbiota composition, SCFA profiles, as well as intestinal, systemic, and neuroinflammation. It was hypothesized that dark red kidney beans, with richer polyphenolic profiles, would confer stronger health benefits compared to white beans. Our findings support this hypothesis: while both bean varieties significantly altered the gut microbiota and elevated SCFA production, only dark red beans improved colonic and hippocampal inflammation. These findings suggest that bean variety influences the extent to which microbiota remodelling and SCFA production may translate into improvements in host inflammatory outcomes. However, given the study design, we cannot establish whether inflammatory improvements were directly driven by microbiota or SCFA changes, and it is possible that other bean-derived components independently contributed to these effects.

Beans are rich sources of fermentable NDCs and phenolic compounds that selectively stimulate SCFA-producing bacteria [45,48,52,53,100]. In line with this, both bean diets significantly modified the gut microbial ecosystem in the context of high-fat feeding. Bean supplementation enriched genera commonly linked with SCFA production [101,102,103], barrier integrity [104], and anti-inflammatory signalling [105,106], such as *Prevotella*, *Lactobacillus*, *Ruminococcus*, and *Muribaculaceae* (*S24-7*), while reducing taxa associated with dysbiosis and obesity-related intestinal barrier permeability defects, such as *rc4-4, Mucispirillum*, and *TM7* [107,108,109]. While there were no significantly different taxa between bean types, HF + WK had decreased *Alistipes*, while HF + DK had increased *Dorea* compared to HF and increased *Papillibacter* compared to BD. The reduction in *Alistipes*, mainly *Alistipes finegoldii* and *Alistipes massiliensis* in HF + WK, and increases in *Dorea longicatena* and *Papillibacter cinnamivorans* in HF + DK may partly contribute to the subtle differences in SCFA production and inflammatory profiles observed between the bean groups, as these species have been associated with SCFA generation and improved metabolic health outcomes [110,111,112,113,114]. This interpretation is supported by correlation analyses showing that *Dorea* abundance was positively associated with cecal acetic acid concentrations, while *Papillibacter* abundance was positively associated with cecal butyric acid, thus linking these taxa to microbial fermentation. Although genus-level differences were modest, they may still influence downstream microbial functions, as bacteria differ in their fermentative capacities and metabolic end products [101,102,103,110,111,112,113,114]. Thus, even subtle compositional shifts may contribute to the distinct SCFA and inflammatory responses observed between bean varieties. These changes align with prior work showing that pulse diets can enhance microbial diversity and select for SCFA-producing bacteria [53,100,115,116], reinforcing the concept that beans contribute to a healthier microbial profile under obesogenic conditions.

Interestingly, *Anaeroplasmataceae gut* (class Mollicutes, now often referred to as Anaeroplasma) were absent in BD and HF groups but appeared in both bean-supplemented groups. Their abundance was positively correlated with colonic IL-10, suggesting a role in anti-inflammatory signalling at the intestinal barrier. Although rarely mentioned in obesity studies, recent work indicates potential protective roles for *Anaeroplasmataceae* against barrier dysfunction and colorectal cancer [116,117]. Importantly, members of this family are known to ferment carbohydrates, and they also require sterols for membrane integrity [118]. Beans are a source of phytosterols; it is likely that bean consumption could create a sterol-rich environment, favouring this taxon. This may partially explain the expansion of this taxon exclusively within the bean groups [25,119]. These observations suggest that bean-derived substrates, including fermentable carbohydrates, sterols, and polyphenols, may create a distinct environment that selectively supports taxa not present under BD or HF conditions, contributing to the distinct metabolic and immunomodulatory environment observed in bean-fed mice.

Given these compositional shifts and their links to fermentation, we next examined the impact of bean diets on predicted and measured SCFA production. Predicted functional profiling indicated that kidney bean supplementation modulated microbial fermentation pathways under high-fat conditions, with distinct effects depending on bean variety. MetaCyc analysis showed that dark red kidney bean supplementation enriched predicted fermentative routes converging on acetate production through terminal reactions mediated by acetate kinase [93], including mixed acid fermentation, pyruvate fermentation to acetate and lactate, and acetylene degradation. These mice also showed enrichment of predicted homolactic fermentation and acetate-producing pathways, suggesting greater availability of lactate and acetate for microbial cross-feeding that could support enhanced butyrate and overall SCFA production [29].

Consistent with these predicted functional shifts, both bean diets increased acetic and butyric acid concentrations alongside elevated predicted acetate kinase abundance, while dark red kidney beans uniquely enriched predicted enzymes involved in butyrate biosynthesis. Fecal acetic acid levels were also higher in mice fed dark beans compared to white beans, suggesting that although both bean varieties increase fermentative capacity, dark red beans may promote a more efficient conversion of fermentable substrates into butyrate and acetate. Butyrate is known to exert anti-inflammatory effects through histone deacetylase inhibition and suppression of NF-κB signalling [117,118]. Accordingly, this functional enrichment may help explain why higher butyric acid levels coincided with increased colonic IL-10 (in all groups) and with reduced serum TNF-α uniquely in HF + DK fed mice, supporting a link between SCFA metabolism and improvements in inflammation. Enhanced fermentation in the bean groups may be related to their capacity to inhibit α-amylase enzymes, leading to increased delivery of NDCs and resistant starch to the colon and promoting microbial fermentation [119]. These results align with previous bean supplementation studies that show enhanced SCFA production [45,49,120,121].

KEGG pathway analysis supported these metabolite-level findings by showing that bean supplementation enhanced predicted microbial pathways involved in biosynthesis and genomic maintenance pathways. These included pyrimidine metabolism, ribosomal pathways, and amino sugar and nucleotide sugar metabolism, suggesting a more metabolically active microbial community. Similar measured and predicted functional enrichments have been reported in other bean supplementation studies [48,50,122,123]. In contrast, HF feeding alone produced only minor functional shifts from BD, indicating that beans were the primary drivers of functional microbial remodelling. Reductions in two-component systems, bacterial chemotaxis and flagellar assembly in bean-fed mice may also reflect a shift away from stress-responsive, virulence, or pro-inflammatory microbial behaviours [124]. Collectively, these functional predictions complement the observed SCFA profiles and support the conclusion that kidney beans enhance microbial fermentative capacity and reduce pro-inflammatory functional profiles, with dark red beans exerting the strongest effects.

Since the role of SCFAs in maintaining barrier integrity and regulating colonic inflammation is well established [125], we next examined colonic outcomes. HF feeding reduced cecum weight, content, and goblet cell numbers, consistent with impaired fermentation and reduced intestinal mass previously reported with obesogenic diets [126,127]. Bean supplementation prevented these changes, which is in line with enhanced microbial fermentation and SCFA-mediated stimulation of mucus production [128,129], although colon weight and serum LBP were unchanged. Because SCFAs regulate inflammation, we next investigated colonic cytokine and receptor expression. Gene expression data provided further evidence of colonic benefits, with TNF-α expression being significantly reduced in the HF + DK group. In contrast, IL-1β were elevated in all high-fat groups, indicating persistent inflammatory signalling under obesogenic conditions. Interestingly, GPR43 expression was lowest in the HF + DK groups, which correlated with reduced colonic TNF-α in the HF + DK group. Since GPR43 expression has been linked to inflammatory tone and varies across tissues [99,130,131,132,133], its downregulation here may reflect an adaptive response in a less inflamed colonic environment. In support of this, a study supplementing rats with black beans also found a decrease in colonic GPR43, which was associated with reduced systemic inflammation (as measured by serum lipopolysaccharide), supporting the possibility that increased GPR43 expression may be indicative of an inflammatory state in our study [49].

High-fat feeding promoted a systemic pro-inflammatory shift, at least as reflected by reduced circulating IL-10, consistent with increased inflammation often observed in obesity [134,135]. Bean supplementation limited IL-10 reductions, supporting prior reports that pulses and polyphenol-rich foods enhance anti-inflammatory signalling [44,50,100,121,136,137]. RANTES tended to be higher in the BD group compared to HF, which was an unexpected result as RANTES is generally higher in serum and adipose tissue in those with obesity [138,139]. Despite that, studies found that physiological levels of RANTES can support insulin signalling and a deficiency can exacerbate high-fat diet-induced inflammation [140,141]. Bean variety effects were also observed such that PAI-1, typically elevated in obesity and type 2 diabetes [142,143,144], was partially normalized with beans. Further, white beans blunted the rise in serum leptin, indicating possible protection from leptin resistance [145], while dark red beans reduced resistin, consistent with anti-inflammatory activity. GIP was reduced across all high-fat-fed groups, which contrasts with reports of elevated GIP under chronic high-fat feeding [146,147,148]. This may reflect an adaptive response, as chronic lowering of GIP is seen with fibre consumption and its reduction is associated with alleviation of diet-induced obesity and insulin resistance [149,150]. Together, these findings indicate that beans attenuated HF-induced systemic inflammation and modulated metabolic hormone signalling, potentially through SCFA- and polyphenol-driven mechanisms, although this would need to be directly tested. The differing effects of white and dark red beans on systemic markers such as leptin and resistin further support the idea that bean varieties differentially influence host inflammatory and metabolic signalling [25,30]. These differences may reflect cultivar-specific interactions between polyphenols, microbial metabolites, and host immune pathways, consistent with the stronger anti-inflammatory phenotype observed in the dark red bean group.

To determine whether the effects of kidney bean consumption extend beyond the gut and systemic inflammation, we examined the dorsal hippocampus, a region involved in memory [151]. In the context of obesity, impairments in hippocampal-dependent cognition and accompanying structural alterations are associated with altered stress and reward pathways, and with depressive-like symptoms [23,24,58,59,60]. HF feeding elevated IL-6 expression, which remained high in HF + WK, but was partially normalized in HF + DK mice, indicating stronger protection by dark red beans. This coincided with modestly elevated NF-κB expression in HF + WK, which could be related to the slight but non-significant elevations in serum LBP and hippocampal TLR4 expression [152]. These patterns suggest that the neuroimmune environment remained more activated in HF + WK mice, whereas dark red beans provided greater attenuation of inflammatory signalling. Given that dark red beans, and consequently the HF + DK diet, contained higher total phenolic content in this study (Tables S1 and Table 1), it is plausible that differences in polyphenolic profiles contributed to these effects observed in HF + DK. Polyphenols are known to interact with both microbial and host pathways, including modulation of microglial activation, suppression of pro-inflammatory cytokine signalling, and attenuation of oxidative stress through reactive oxygen species scavenging [136,153,154,155]. Many dietary polyphenols reach the colon largely intact and are transformed by the gut microbiota into bioactive metabolites that influence host immune and neuroinflammatory responses [39,42,43,44,156]. Therefore, higher or distinct polyphenol content in dark red beans may have contributed to reduced neuroinflammation relative to white beans.

Bean diets slightly but non-significantly increased occludin and, in the case of HF + WK, ZO-1, suggested improved BBB integrity. The HF + WK group showed a positive correlation between ZO-1 and MCT-1, consistent with the hypothesis that SCFAs may support BBB integrity through transporter-mediated uptake [157]; however, this evidence is only supportive and requires direct testing. Beyond the effects of SCFAs, interactions between bean polyphenols and non-digestible polysaccharides may further enhance microbial fermentation and SCFA availability [156,158], providing an additional pathway through which dark red beans exert greater neuroprotective effects. These results align with evidence that NDCs and phytochemicals can modulate gut–brain interactions to increase BBB integrity, reduce microglial activation and attenuate neuroinflammation [21,154,155,157,159,160], and suggest that bean seed coat colour influences the extent to which these benefits are seen in the context of obesity [152].

This study has limitations that should be considered. 16s rRNA sequencing and PICRUSt2 predictions provide insight into composition and inferred function but cannot fully capture strain-level variation or direct metabolic activity; metagenomic and metabolomic approaches are needed. Cytokine and gene expression were assessed using targeted transcript- and protein-level approaches, which may not fully capture broader transcriptional changes across inflammatory pathways; future studies incorporating broader transcriptomic approaches, such as RNA sequencing or expanded inflammatory gene panels, could further expand upon these findings. The use of a single sex and mouse strain also limits generalizability; however, the C57Bl/6 mouse model supplemented with high-fat diets reproduces many aspects of the obese phenotype in humans relevant to diet-induced metabolic and inflammatory dysfunction, supporting the translational relevance of these findings [161]. A key strength of this work is the inclusion of soluble fibre in the control diets, which allowed us to isolate bean-specific effects beyond those of fermentable fibre. Additional strengths include testing two bean types within the same market class (e.g., kidney beans), differing by seed coat colour and phenolic profiles, as well as the comprehensive assessment of gut, systemic, and brain outcomes, and the integration of microbial, biochemical, and molecular endpoints to capture host-microbiota interactions.

## 5. Conclusions

Collectively, this study demonstrates that kidney bean supplementation reshaped the gut microbiota, enhanced SCFA production, and attenuated intestinal, systemic, and neuroinflammatory disturbances in the context of high-fat feeding. While both bean types conferred benefits, dark red kidney beans exerted stronger anti-inflammatory effects, which is consistent with their higher phenolic content. By using controls with matched ratios of soluble to insoluble fibre, this study distinguishes bean-specific effects from those of fermentable fibre, providing a rigorous evaluation of seed coat colour-driven outcomes. These findings highlight bean variety as an important determinant of microbiota-mediated health benefits and reinforce the potential of pulse interventions tailored by seed coat colour for obesity-associated inflammation.

## Figures and Tables

**Table 1:** Experimental diet composition.

Nutrient (g/kg)	BD TD.180554	HF TD.180557	HF + WK TD.180558	HF + DK TD.180556
Casein	200.0	265.0	225.0	226.22
L-Cystine	3.0	4.0	4.0	4.0
Corn Starch	377.486	0.0	0.0	0.0
Maltodextrin	132.0	160.0	94.72	91.3072
Sucrose	100.0	92.586	92.586	92.586
Lard	0.0	310.0	310.0	310.0
Soybean Oil	70.0	30.0	28.33	28.2728
Cellulose	50.0	50.0	17.75	18.5
Pectin	20.0	20.0	9.2	10.7
Mineral Mix, AIN-93G-MX (94046)	35.0	48.0	48.0	48.0
Vitamin Mix, AIN-93-VX (94047)	10.0	14.0	14.0	14.0
Calcium Phosphate, dibasic	0.0	3.4	3.4	3.4
Choline Bitartrate	2.5	3.0	3.0	3.0
TBHQ, antioxidant	0.014	0.014	0.014	0.014
White Kidney Bean Powder	0.0	0.0	150.0	0.0
Dark Red Kidney Bean Powder	0.0	0.0	0.0	150.0
TPC (mg GAE/g diet)	0.24 ± 0.005 ^c^	0.25 ± 0.01 ^c^	0.34 ± 0.009 ^b^	0.44 ± 0.009 ^a^
**Contribution of total calories from each macronutrient**				
Protein (% kcal)	19.2	18.4	18.5	18.5
Carbohydrate (% kcal)	63.2	21.1	20.7	20.8
Fat (% kcal)	17.6	60.5	60.8	60.7
Energy Density (kcal/g)	3.7	5.1	5.1	5.1

Formulations of the basal diet (BD), high-fat diet (HF), HF supplemented with 15% white kidney bean powder (HF + WK), and HF supplemented with 15% dark red kidney bean powder (HF + DK). Diets were prepared according to the AIN-93G formulation, with corn oil substituted for soybean oil, and 2% pectin added. TPC was determined using Folin–Ciocalteu method and was analyzed by ANOVA. Values are presented as mean ± SEM. Groups not sharing a lowercase letter are significantly different (*p* < 0.05). TBHQ = t-butylhydroquinone; GAE = gallic acid equivalents; TPC = Total phenolic content.

**Table 2:** Relative abundance (%) of cecal microbiota in mice fed experimental diets.

	BD	HF	HF + WK	HF + DK
p__Firmicutes	31.449 ± 2.1	34.233 ± 1.838	27.984 ± 1.195 #	32.469 ± 1.514
c__Bacilli;o__Bacillales;f__Bacillaceae;__	0.076 ± 0.041	0 ± 0	0 ± 0	0 ± 0
c__Bacilli;o__Lactobacillales;f__Lactobacillaceae;g__Lactobacillus	0.266 ± 0.135	0.06 ± 0.012	0.23 ± 0.056 #	0.251 ± 0.102 #
c__Clostridia;__;__;__	0 ± 0	0.084 ± 0.015 *	0.038 ± 0.011 *#	0.077 ± 0.019 *
c__Clostridia;o__Clostridiales;__;__	3.425 ± 0.702	3.4 ± 0.423	4.543 ± 0.664	3.451 ± 0.321
c__Clostridia;o__Clostridiales;f__;g__	2.775 ± 0.691	1.902 ± 0.391	0.962 ± 0.101 *#	1.28 ± 0.25
c__Clostridia;o__Clostridiales;f__Lachnospiraceae;__	8.297 ± 0.806	7.543 ± 1.547	7.259 ± 1.035	10.065 ± 0.896
c__Clostridia;o__Clostridiales;f__Lachnospiraceae;g__Clostridium	6.907 ± 1.347	5.582 ± 1.12	5.553 ± 0.561	6.738 ± 0.848
c__Clostridia;o__Clostridiales;f__Lachnospiraceae;g__Defluviitalea	0.415 ± 0.067	0.8 ± 0.15	0.359 ± 0.028 #	0.411 ± 0.064 #
c__Clostridia;o__Clostridiales;f__Lachnospiraceae;g__Dorea	0.104 ± 0.021	0.15 ± 0.022	0.165 ± 0.019	0.248 ± 0.027 *#
c__Clostridia;o__Clostridiales;f__Lachnospiraceae;g__Lachnospira	0.126 ± 0.061	0.86 ± 0.27 *	0.148 ± 0.063 #	0.083 ± 0.028 #
c__Clostridia;o__Clostridiales;f__Lachnospiraceae;g__Ruminococcus	0.008 ± 0.008	0.011 ± 0.007	0.615 ± 0.171 *#	0.386 ± 0.071 *#
c__Clostridia;o__Clostridiales;f__Peptococcaceae;g__rc4-4	0.985 ± 0.216	0.882 ± 0.164	0.373 ± 0.058 *#	0.332 ± 0.043 *#
c__Clostridia;o__Clostridiales;f__Peptostreptococcaceae;__	0 ± 0	0.689 ± 0.404	0.148 ± 0.113	0.02 ± 0.02
c__Clostridia;o__Clostridiales;f__Ruminococcaceae;__	3.922 ± 1.105	2.584 ± 0.4	1.927 ± 0.228	2.11 ± 0.264
c__Clostridia;o__Clostridiales;f__Ruminococcaceae;g__	2.052 ± 0.265	4.471 ± 1.037	2.737 ± 0.191	2.934 ± 0.309
c__Clostridia;o__Clostridiales;f__Ruminococcaceae;g__Clostridium	1.062 ± 0.108	2.812 ± 0.371 *	1.747 ± 0.27 #	2.165 ± 0.282 *
c__Clostridia;o__Clostridiales;f__Ruminococcaceae;g__Oscillospira	0.529 ± 0.057	1.061 ± 0.162 *	0.411 ± 0.048 #	0.823 ± 0.208
c__Clostridia;o__Clostridiales;f__Ruminococcaceae;g__Papillibacter	0.033 ± 0.02	0 ± 0	0.093 ± 0.031 #	0.229 ± 0.146 *#
c__Clostridia;o__Clostridiales;f__Ruminococcaceae;g__Sporobacter	0.458 ± 0.051	1.334 ± 0.318 *	0.669 ± 0.106	0.858 ± 0.116 *
p__Bacteroidetes	59.365 ± 2.108	55.35 ± 1.787	61.383 ± 1.731 #	58.777 ± 1.252
c__Bacteroidia;o__Bacteroidales;__;__	0.572 ± 0.084	0.589 ± 0.247	0.812 ± 0.096 #	0.631 ± 0.164
c__Bacteroidia;o__Bacteroidales;f__Bacteroidaceae;g__Bacteroides	23.623 ± 2.256	27.343 ± 1.492	19.546 ± 1.446 #	18.766 ± 0.984 #
c__Bacteroidia;o__Bacteroidales;f__Porphyromonadaceae;g__Parabacteroides	0.85 ± 0.138	0.674 ± 0.064	0.631 ± 0.124	0.4 ± 0.032 *#
c__Bacteroidia;o__Bacteroidales;f__Prevotellaceae;g__Prevotella	3.112 ± 0.488	0.366 ± 0.13 *	6.454 ± 0.785 *#	3.786 ± 0.741 #
c__Bacteroidia;o__Bacteroidales;f__Rikenellaceae;__	2.911 ± 0.513	4.315 ± 0.85	9.705 ± 0.893 *#	9.643 ± 1.044 *#
c__Bacteroidia;o__Bacteroidales;f__Rikenellaceae;g__Alistipes	8.843 ± 1.032	11.491 ± 1.044	7.397 ± 0.591 #	9.092 ± 0.996
c__Bacteroidia;o__Bacteroidales;f__S24-7;g__	19.45 ± 1.557	10.569 ± 0.923 *	16.837 ± 1.54 #	16.456 ± 1.499 #
p__Deferribacteres	4.053 ± 0.411	7.965 ± 0.928 *	2.997 ± 0.545 #	3.681 ± 0.455 #
c__Deferribacteres;o__Deferribacterales;f__Deferribacteraceae;g__Mucispirillum	4.053 ± 0.411	7.965 ± 0.928 *	2.997 ± 0.545 #	3.681 ± 0.455 #
p__Proteobacteria	2.133 ± 0.345	0.934 ± 0.244 *	1.746 ± 0.295 #	1.156 ± 0.241 *
p__Proteobacteria;c__Alphaproteobacteria;__;__;__	1.663 ± 0.34	0.917 ± 0.24	1.111 ± 0.291	0.795 ± 0.217
p__Proteobacteria;c__Alphaproteobacteria;o__RF32;f__;g__	0.294 ± 0.191	0 ± 0	0.299 ± 0.15 #	0.09 ± 0.057
p__Proteobacteria;c__Betaproteobacteria;o__Burkholderiales;__;__	0.174 ± 0.059	0.017 ± 0.008 *	0.335 ± 0.051 *#	0.27 ± 0.055 #
p__Verrucomicrobia	1.635 ± 0.279	0.68 ± 0.355 *	1.037 ± 0.308	0.513 ± 0.185 *
c__Verrucomicrobiae;o__Verrucomicrobiales;f__Verrucomicrobiaceae;g__Akkermansia	1.635 ± 0.279	0.68 ± 0.355 *	1.037 ± 0.308	0.513 ± 0.185 *
p__Cyanobacteria	1.319 ± 0.47	0.443 ± 0.176 *	0.863 ± 0.2	0.521 ± 0.276 *
c__4C0d-2;o__YS2;f__;g__	1.319 ± 0.47	0.443 ± 0.176 *	0.863 ± 0.2	0.521 ± 0.276 *
p__Tenericutes	0 ± 0	0 ± 0	3.844 ± 1.001 *#	2.743 ± 0.539 *#
c__Mollicutes;o__Anaeroplasmatales;f__Anaeroplasmataceae;g__gut	0 ± 0	0 ± 0	3.844 ± 1.001 *#	2.743 ± 0.539 *#
p__TM7	0.043 ± 0.019	0.392 ± 0.136 *	0.142 ± 0.027 *	0.136 ± 0.034 *
c__TM7-3;o__CW040;f__F16;g__	0.043 ± 0.019	0.392 ± 0.136 *	0.142 ± 0.027 *	0.136 ± 0.034 *

Relative abundance (mean ± SEM) of cecal bacterial taxa at the phylum level (highlighted in grey rows), and corresponding order (o), family (f), and/or genus (g) with abundance greater than 0.03% in at least one group. Values marked with an asterisk (*) differ from BD, and values marked with a (#) symbol differ from HF (*p* < 0.05).

**Table 3:** Metabolic health parameters in serum and epididymal adipose tissue in mice fed experimental diets.

Sample Type	Marker	BD	HF	HF + WK	HF + DK	Statistics
**Serum**	**Adipose- and Endothelial-derived Hormones (pg/mL)**					
	Leptin	4656 ± 958.0 ^b^	14,662 ± 4306 ^a^	12191 ± 3536 ^ab^	19,473 ± 4917 ^a^	F (3, 40) = 5.434, *p* = 0.0031
	Resistin	30,018 ± 1046	37,640 ± 2143	35,263 ± 3802	30,652 ± 6398	H(3) = 5.488, *p* = 0.1394
	PAI-1	966.6 ± 48.97 ^a^	776.7 ± 42.69 ^b^	830.9 ± 45.44 ^ab^	817.3 ± 30.26 ^ab^	F (3, 41) = 3.854, *p* = 0.0161
	**Gut-derived peptide hormones (pg/mL)**					
	GIP	80.71 ± 2.208 ^a^	70.14 ± 2.495 ^b^	68.49 ± 2.097 ^b^	67.85 ± 2.252 ^b^	H(3) = 15.08, *p* = 0.0018
	Ghrelin	2474 ± 136.2	2227 ± 226.5	2087 ± 197.4	1779 ± 243.7	F (3, 41) = 2.146, *p* = 0.1091
	**Biomarkers of Blood Glucose Regulation and Insulin Resistance**					
	Glucose (mmol/L)	8.75 ± 0.2811 ^b^	10.08 ± 0.1896 ^a^	10.10 ± 0.2777 ^a^	10.30 ± 0.2585 ^a^	F (3, 41) = 7.944, *p* = 0.0003
	Insulin (pg/mL)	1549 ± 151.3	1823 ± 108.2	1850 ± 114.6	1841 ± 123.9	F (3, 41) = 1.347, *p* = 0.2724
	HOMA-IR	3.380 ± 0.3010 ^b^	4.975 ± 0.3583 ^a^	5.073 ± 0.4106 ^a^	5.178 ± 0.4468 ^a^	F (3, 40) = 4.843, *p* = 0.0057
**Adipose**	**Adipose- and Endothelial-derived Hormones (pg/mL)**					
	Leptin	9880 ± 1324 ^b^	15,901 ± 1595 ^a^	16,700 ± 1514 ^a^	18,551 ± 1859 ^a^	F (3, 40) = 5.60, *p* = 0.0026
	Resistin	85,102 ± 6574 ^a^	66,356 ± 7699 ^ab^	65,244 ± 7449 ^ab^	52,935 ± 7037 ^b^	F (3, 41) = 3.655, *p* = 0.0201
	PAI-1	634.0 ± 71.30	727.1 ± 80.36	824.4 ± 54.16	810.3 ± 61.29	F (3, 41) = 2.481, *p* = 0.0745

Concentrations of adipose-, endothelial-, and gut-derived hormones, blood glucose, insulin, and HOMA-IR were measured in fasted serum collected at week 7 and adipose tissue after 9 weeks of dietary intervention. Values are presented as mean ± SEM. Groups not sharing a lowercase letter are significantly different (*p* < 0.05). Abbreviations: GIP = Glucose-dependent insulinotropic polypeptide; PAI-1 = plasminogen activator inhibitor-1; HOMA-IR = Homeostatic Model Assessment of Insulin Resistance. BD = Basal diet, HF = High-fat diet, HF + WK = HF + white kidney bean, HF + DK = HF + dark red kidney bean.

## Data Availability

The data presented in this study are available on request from the corresponding author due to the data being part of an ongoing study.

## Supplementary Materials

The following supporting information can be downloaded at: https://www.mdpi.com/article/10.3390/nu18030461/s1, Figure S1: Effects of bean supplementation in a high-fat diet on body weight, diet intake, and body composition. Figure S2. Overlapping genus-level microbial shifts between dietary groups. Figure S3. Predicted functional contributions of *Prevotella* spp. across diet groups. Table S1: Cooked white and dark red kidney bean powder proximate analysis and fiber content. Table S2: PCR Primer Sequences. Table S3. Post hoc comparisons of weekly body weight between dietary groups. Table S4: Adipose and liver weights in mice fed experimental diets. Table S5. Differentially abundant bacterial taxa across dietary groups. Table S6. Species relative abundance (%) of cecal microbiota in mice fed experimental diets. Table S7. Statistical comparisons for taxon-function heatmap of top contributing microbial pathways. Table S8. Serum inflammatory cytokine and chemokine profiles.
