# Supplementary material for: Varietal Differences in Kidney Beans Modulate Gut Microbiota and Inflammation During High-Fat Diet-Induced Obesity in Male Mice
Source: Nutrients. 2026 Jan 30;18(3):461. doi: 10.3390/nu18030461 (PMC12899357; doi:10.3390/nu18030461)
Supplement: Supplementary file 1 [file nutrients-18-00461-s001.zip › Supplementary Figure Captions.pdf]

Supplementary Figure Captions:

**Figure S1: Effects of bean supplementation in a high-fat diet on body weight, diet intake, and body composition.**
(A) Percent change in body weight over time (weeks), final measurement taken at week 8.5 (plotted as week 9 for visualization). Body weight differed significantly across diet groups during the intervention; full weekly post hoc comparisons are provided in Supplementary table S3. (B) Final body weight (g). (C) Dietary intake (kcal/mouse/day) over time. (D) Dietary intake (g/mouse/day) over time. Mice in BD group consumed significantly more grams of diet per day than all HF-based groups across all timepoints ($P<0.05$). (E) Lean mass (g) at weeks 2 and 6. (F) Fat mass (g) at weeks 2 and 6. (G) Adiposity (Fat mass as % of body weight) at week 6. Data are presented as mean ± SEM. Groups not sharing a lowercase letter are significantly different ($p<0.05$). BD = basal diet; BD = Basal diet, HF = High-fat diet, HF+WK = HF + white kidney bean, HF+DK = HF + dark red kidney bean.

**Figure S2. Overlapping genus-level microbial shifts between dietary groups.**
Venn diagrams illustrate the number of genera with significantly increased (top row) or decreased (bottom row) relative abundance in response to dietary interventions compared to either basal diet (BD; left column) or high-fat diet (HF; right column). Numbers reflect the unique and shared genera altered by each group. BD = Basal diet, HF = High-fat diet, HF+WK = HF + white kidney bean, HF+DK = HF + dark red kidney bean.

**Figure S3. Predicted functional contributions of *Prevotella* spp. across diet groups.**
BURRITO visualization of *Prevotella* spp. functional potential in the cecal microbiota based on PICRUSt2 predictions. Upper panels illustrate the taxonomic hierarchy leading to *Prevotella* and its contributions to major KEGG Level 1 functional categories. Lower panels display (left) the relative abundance of *Prevotella* in each sample and (right) the proportional distribution of its predicted functional pathways across individual mice in each diet group. BD = Basal diet, HF = High-fat diet, HF+WK = HF + white kidney bean, HF+DK = HF + dark red kidney bean.
