# Supplementary figures and images for: Varietal Differences in Kidney Beans Modulate Gut Microbiota and Inflammation During High-Fat Diet-Induced Obesity in Male Mice
Source: Nutrients. 2026 Jan 30;18(3):461. doi: 10.3390/nu18030461 (PMC12899357; doi:10.3390/nu18030461)

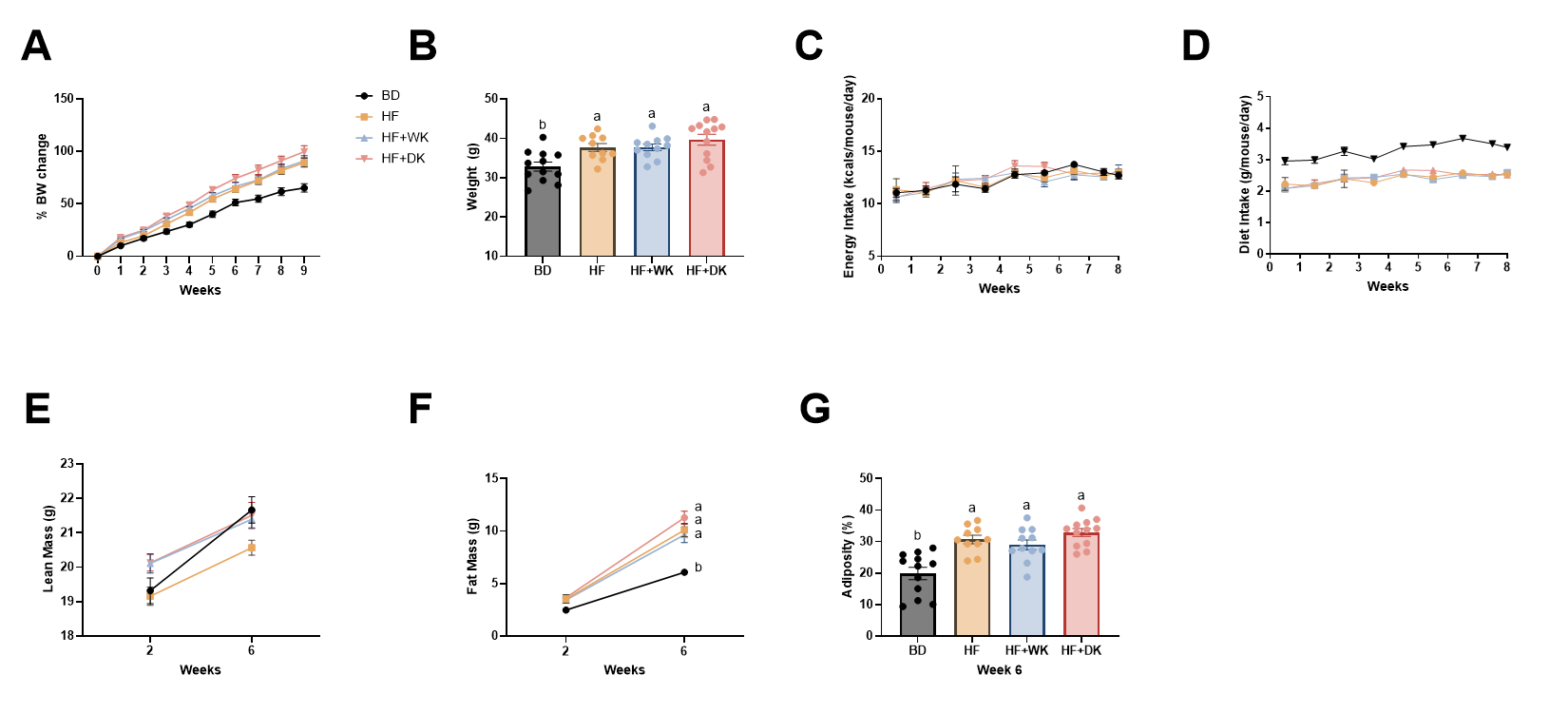

Supplement: Supplementary file 1 [file nutrients-18-00461-s001.zip › FigureS1.png]

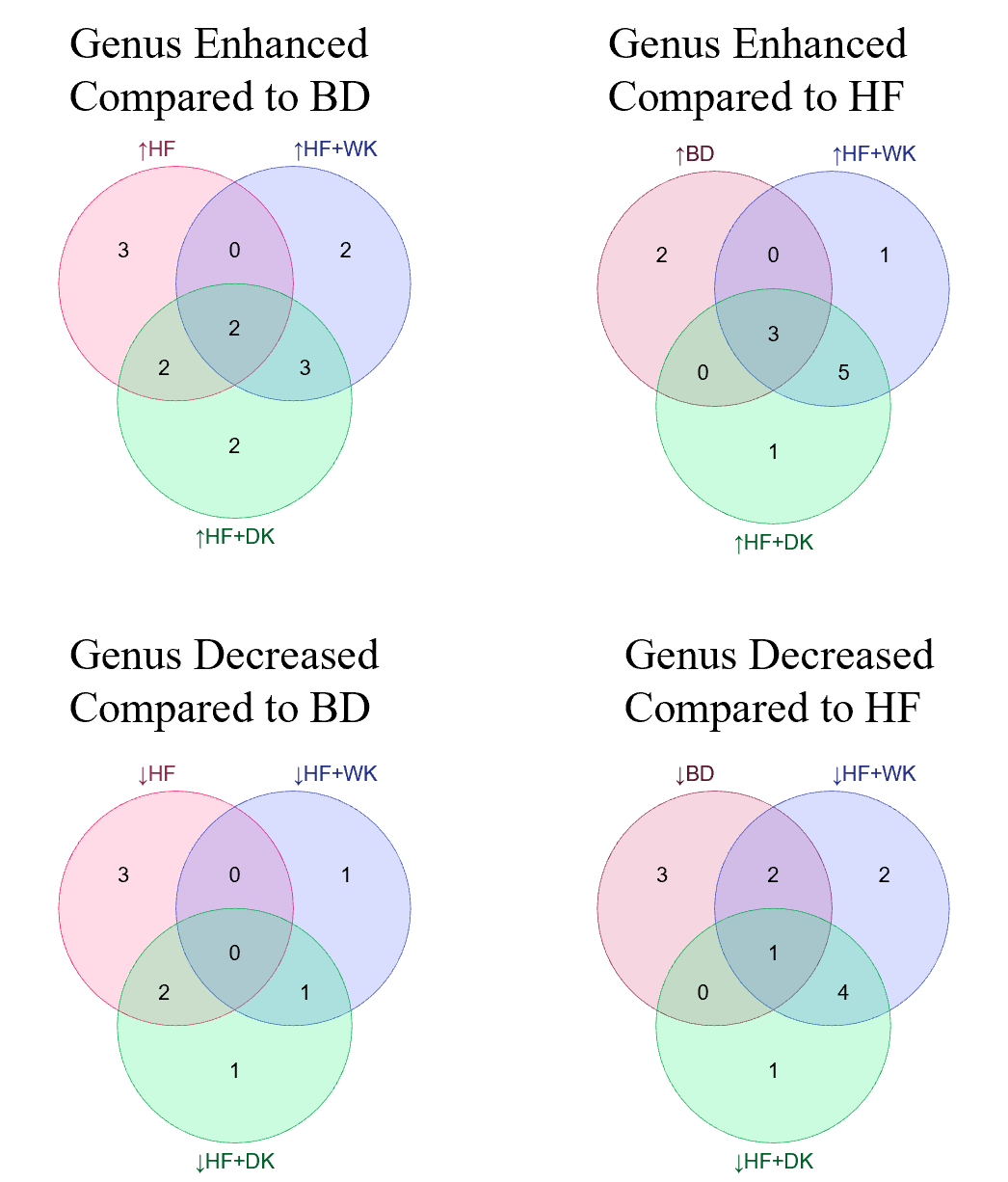

Supplement: Supplementary file 1 [file nutrients-18-00461-s001.zip › FigureS2.png]
